# Downregulation of miR-133a-3p promotes prostate cancer bone metastasis via activating PI3K/AKT signaling

**DOI:** 10.1186/s13046-018-0813-4

**Published:** 2018-07-18

**Authors:** Yubo Tang, Jincheng Pan, Shuai Huang, Xinsheng Peng, Xuenong Zou, Yongxiang Luo, Dong Ren, Xin Zhang, Ronggang Li, Peiheng He, Qingde Wa

**Affiliations:** 1 Department of Pharmacy, The First Affiliated Hospital of Sun Yat-Sen University, Guangzhou, 510080 China; 2 Guangdong Provincial Key Laboratory of Orthopaedics and Traumatology, the First Affiliated Hospital of Sun Yat-sen University, Guangzhou, 510080 China; 3 Department of Urology Surgery, the First Affiliated Hospital of Sun Yat-sen University, Guangzhou, 510080 China; 4 Department of Orthopaedic Surgery, the Second Affiliated Hospital of Guangzhou Medical University, Guangzhou, 510260 China; 5 Department of Orthopaedic Surgery, the First Affiliated Hospital of Sun Yat-sen University, 58# Zhongshan 2rd Road, Guangzhou, 510080 Guangdong Province China; 6 Department of biomedical engineering, health science center, Shenzhen University, Shenzhen, 518060 China; 7 Department of Pathology, Jiangmen Central Hospital, Affiliated Jiangmen Hospital of Sun Yat-sen University, Jiangmen, 529030 China; 8 Department of Orthopaedic Surgery, the Affiliated Hospital of Zunyi Medical college, 149 Dalian Road, Zunyi, 563003 Guizhou Province China

**Keywords:** miR-133a-3p, Cytokine receptor, Bone metastasis, PI3K/AKT signaling pathway, Prostate cancer

## Abstract

**Background:**

Bone metastasis is a leading cause of morbidity and mortality in advanced prostate cancer (PCa). Downexpression of miR-133a-3p has been found to contribute to the progression, recurrence and distant metastasis in PCa. However, clinical significance of miR-133a-3p in bone metastasis of PCa, and the biological role of miR-133a-3p and its molecular mechanisms underlying bone metastasis of PCa remain unclear.

**Methods:**

miR-133a-3p expression was evaluated in 245 clinical PCa tissues by real-time PCR. Statistical analysis was performed to evaluate the clinical correlation between miR-133a-3p expression and clinicopathological features, and overall and bone metastasis-free survival in PCa patients. The biological roles of miR-133a-3p in the bone metastasis of PCa were investigated both in vitro and in vivo. Bioinformatics analysis, real-time PCR, western blot and luciferase reporter analysis were applied to demonstrate the relationship between miR-133a-3p and its potential targets. Western blotting and luciferase assays were examined to identify the underlying pathway involved in the anti-tumor role of miR-133a-3p. Clinical correlation of miR-133a-3p with its targets was verified in human PCa tissues.

**Results:**

miR-133a-3p expression is reduced in PCa tissues compared with the adjacent normal tissues and benign prostate lesion tissues, particularly in bone metastatic PCa tissues. Low expression of miR-133a-3p is significantly correlated with advanced clinicopathological characteristics and shorter bone metastasis-free survival in PCa patients by statistical analysis. Moreover, upregulating miR-133a-3p inhibits cancer stem cell-like phenotypes in vitro and in vivo, as well as attenuates anoikis resistance in vitro in PCa cells. Importantly, administration of agomir-133a-3p greatly suppresses the incidence of PCa bone metastasis in vivo. Our results further demonstrate that miR-133a-3p suppresses bone metastasis of PCa via inhibiting PI3K/AKT signaling by directly targeting multiple cytokine receptors, including EGFR, FGFR1, IGF1R and MET. The negative clinical correlation of miR-133a-3p with EGFR, FGFR1, IGF1R, MET and PI3K/AKT signaling activity is determined in clinical PCa tissues.

**Conclusion:**

Our results unveil a novel mechanism by which miR-133a-3p inhibits bone metastasis of PCa, providing the evidence that miR-133a-3p may serve as a potential bone metastasis marker in PCa, and delivery of agomir-133a-3p may be an effective anti-bone metastasis therapeutic strategy in PCa.

**Electronic supplementary material:**

The online version of this article (10.1186/s13046-018-0813-4) contains supplementary material, which is available to authorized users.

## Background

Prostate cancer (PCa) is the second common diagnosed cancer in men worldwide and the fifth leading cause of cancer-related deaths [1]. The 5-year relative survival rate of primary PCa patients is > 99%, while that of patients with distant metastasis sites is no more than 30% [2]. Bone is among the most preferential metastatic site of PCa [3]. Once tumor cell metastasis to bone, it will cause several bone- associated complications, including hypercalcemia, intractable pain, fracture, or nerve compression syndrome, contributing to the poor survival in PCa patients [4]. A major challenge for treatment of advanced metastatic disease is due to incomplete understanding of the molecular mechanisms underlying the high avidity of PCa to bone. Therefore, it’s significantly necessary to unveil the molecular mechanism underlying the high bone metastatic propensity of PCa.

Since its initial discovery as a proto-oncogene signaling, the critical roles of the phosphoinositide 3-kinase (PI3K)/Akt Signaling in diverse cellular processes, including cell growth, proliferation and survival, has seized considerable attention [5, 6]. The PI3K family is activated in response to multiple extracellular stimuli, including EGF [7], IGF-1 [8], insulin [9] and CaM [10]. Then, the activated PI3K phosphorylates phosphatidylinositol-3,4-bisphosphate [PI(3,4)P2] and phosphatidylinositol − 3,4,5-trisphosphate [PI(3,4,5)P3], at the 3′-hydroxyl group of the inositol ring of phosphatidylinositol, which further recruits Akt and phosphoinositide-dependent kinases to the plasma membrane, leading to activation of Akt kinase by two phosphorylation sites at Thr 308 and Ser 473 [11, 12]. The activated Akt further phosphorylates multiple downstream effectors, which promotes cells unlimited proliferation and growth [13–15]. Constitutive activation of the PI3K/Akt pathway has been reported to contribute to the pathogenesis of many types of cancer [16]. Furthermore, increasing evidence is accumulating that activity of PI3K/Akt signaling plays a crucial role in the metastasis of cancer. A study by Xue and colleagues has shown that Akt-mediated Twist1 phosphorylation promoted breast cancer lung metastasis [17]. Ectopic expression of cyclin G1 promoted epithelial-mesenchymal transition (EMT) and metastasis of hepatocellular carcinoma cells via enhancing Akt activation-mediated the stabilization of Snail, a critical EMT mediator [18]. Importantly, Ni et al. have reported that PI3K/Akt signaling -mediated stabilization of histone methyltransferase WHSC1 profoundly increased bone metastasis and osteolytic bone lesions in PCa [19]. However, the underlying mechanism responsible for activation of PI3K/Akt signaling in bone metastasis of PCa remains largely unknown.

microRNAs (miRNAs) play important roles in cellular differentiation, proliferation, and embryo development [20], and have been involved in the development, progression and metastasis of cancer [21–23]. An accumulating body of studies has determined the pivotal roles of miRNAs in bone metastasis of PCa [24–28]. miR-133a-3p was one of the frequently deregulated miRNA in cancer [29–31], and low expression of miR-133a-3p has been associated with recurrence and metastasis of PCa [32–36]. However, the clinical significance of miR-133a-3p in the progression and bone metastasis of PCa, and the biological role of miR-133a-3p and its molecular mechanisms underlying bone metastasis of PCa have not been reported. Here we reported that miR-133a-3p was decreased in PCa tissues and further downregulated in bone metastatic PCa tissues, which was positively associated with poor clinicopathological characteristics and bone metastasis-free survival in PCa patients. Moreover, upregulating miR-133a-3p dramatically inhibited cancer stem cells characteristics and anoikis resistance in vitro, and tumorigenesis and bone metastasis in vivo in PCa cells. Our results further demonstrated that miR-133a-3p repressed activity of PI3K/AKT signaling by simultaneously targeting EGFR, FGFR1, IGF1R and MET, which further suppressed bone metastasis of PCa. Therefore, our results clarify the underlying mechanism to determinate the anti-bone metastatic role of miR-133a-3p in PCa.

## Methods

### Cell lines and cell culture

The human PCa cell lines 22RV1, PC-3, VCaP, DU145, LNCaP and normal prostate epithelial cells RWPE-1 were obtained from Shanghai Chinese Academy of Sciences cell bank (China). RWPE-1 cells were grown in defined keratinocyte-SFM (1×) (Invitrogen). PC-3, LNCaP and 22Rv1 cells were cultured in RPMI-1640 medium (Life Technologies, Carlsbad, CA, US) supplemented with penicillin G (100 U/ml), streptomycin (100 mg/ml) and 10% fetal bovine serum (FBS, Life Technologies). DU145 and VCaP cells were grown in Dulbecco’s modified Eagle’s medium (Invitrogen) supplemented with 10% FBS. The C4-2B cell line was purchased from the MD Anderson Cancer Center and maintained in T-medium (Invitrogen) supplemented with 10% FBS. All cell lines were grown under a humidified atmosphere of 5% CO2 at 37 °C.

### Plasmids, transfection and generation of stable cell lines

The human MIR133A gene was PCR-amplified from genomic DNA and cloned into a pMSCV-puro retroviral vector (Clontech, Japan). The 3’UTR of EGFR, FGFR1, IGF1R and MET were PCR-amplified from genomic DNA and cloned into pmirGLO vectors (Promega, USA), and the list of primers used in cloning reactions is shown in Additional file 1: Table S1. AgomiR-133a-3p was synthesized and purified by RiboBio. Cells were treated with MK-2206 (Selleck Chemicals, Houston, TX, USA) at the concentrations (1 μM). Transfection of miRNA, siRNAs, and plasmids was performed using Lipofectamine 3000 (Life Technologies, USA) according to the manufacturer’s instructions.

### RNA extraction, reverse transcription, and real-time RT-PCR

Total RNA from tissues or cells was extracted using the RNA Isolation Kit (Qiagen, USA) according to the manufacturer’s instructions. Messenger RNA (mRNA) were reverse transcribed from total mRNA using the RevertAid First Strand cDNA Synthesis Kit (Thermo Fisher, USA). The random primer was used for reverse transcription of mRNA and the primer for reverse transcription of miRNA were synthesized and purified by RiboBio (Guangzhou, China). Complementary DNA (cDNA) was amplified and quantified on the CFX96 system (BIO-RAD, USA) using iQ SYBR Green (BIO-RAD, USA). The primers are provided in Additional file 2: Table S2. Real-time PCR was performed according to a standard method, as described previously [37]. Primers for U6 and miR-133a-3p were synthesized and purified by RiboBio (Guangzhou, China). U6 or glyceraldehyde-3-phosphate dehydrogenase (GAPDH) was used as the endogenous controls. Relative fold expressions were calculated with the comparative threshold cycle (2^-ΔΔCt^) method.

### Patients and tumor tissues

A total of 225 individual and 20 paired PCa tissues, and 48 benign prostate lesions tissues were obtained during surgery or needle biopsy at The Clinical Biobank of Collaborative Innovation Center for Medical Molecular Diagnostics of Guangdong Province, The Affiliated Jiangmen Hospital of Sun Yat-sen University (Guangdong, China), and the Second Affiliated Hospital of Guangzhou Medical University (Guangdong, China) between January 2008 and December 2016. Patients were diagnosed based on clinical and pathological evidence, and the specimens were immediately snap-frozen and stored in liquid nitrogen tanks. For the use of these clinical materials for research purposes, prior patient’ consents and approval from the Institutional Research Ethics Committee were obtained. The clinicopathological features of the patients are summarized in Additional file 3: Table S3, Additional file 4: Table S4, Additional file 5: Table S5. The median of miR-133a-3p expression in PCa tissues was used to stratify high and low expression of miR-133a-3p.

### miRNA immunoprecipitation

Cells were co-transfected with HA-Ago2, followed by HA-Ago2 immunoprecipitation using anti-HA-antibody. Real-time PCR analysis of the IP material was performed to test the association of the mRNA of EGFR, FGFR1, IGF1R and MET with the RISC complex. The specific processes were performed as previously described [38]. Briefly, Cells (5 × 10^5^) were plated in 60-mm cell culture dishes, proliferating to 60–80% confluence after 24 h of culture, and the pIRESneo-FLAG/HA-Ago2 plasmas were cotransfected into cells using Lipofectamine 3000. After 48-h transfection, cells were washed and lysed in radioimmunoprecipitation buffer (Sigma-Aldrich) containing 10% proteinase inhibitor cocktail (Sigma-Aldrich) and 1 mMphenylmethylsulfonyl fluoride (Sigma-Aldrich). A fraction of the whole cell lysate was used for RNA isolation, and the remaining lysate was subjected to immunoprecipitation (IP) using an antibody against Ago2 (Abcam) or immunoglobulin G (IgG) (Abcam). RNA from whole cell lysates and RNA IP (RIP) fractions was extracted with TRIzol (Life Technologies) according to the manufacturer’s instructions. The relative levels of mRNA were determined using real-time RT-PCR as described above. The relative mRNA enrichment in the RIP fractions was computed based on the ratio of relative mRNA levels in the RIP fractions and the relative mRNA levels in the whole cell lysates.

### Western blot

Western blot was performed according to a standard method, as previously described [39]. Antibodies against Bcl2, Bcl-xl, Surviva and Mcl-1 were purchased from Abcam (Cambridge, USA), and EGFR, FGFR1, IGF1R, MET, p-AKT (S473), p-AKT (T308) and AKT were purchased from Cell Signaling Technology. As a loading control, membranes were stripped and reprobed with an anti-α-tubulin antibody (Sigma-Aldrich, USA).

### Luciferase reporter assay

Cells (4 × 10^4^) were seeded in triplicate in 24-well plates and cultured for 24 h and performed as previously described [40]. Luciferase and Renilla signals were measured 36 h after transfection using a Dual Luciferase Reporter Assay Kit (Promega). The mutations can be designed at any site, but the best one should be around the seed nucleotides in miRNA target tracks. You can design the two pairs of PCR primers, one pair for uspstream fragment, in which the primer for the bottom strand has the nucleotides in the seed region mutated; the other pair for the downstream fragment, in which the 5′ end of primer for the top strand should be overlapping at least 6 nucleotides with the 5′ end of the bottom strand primer for the upstream fragment. Two rounds of PCR are to be run, first round is run to amplify the upstream and downstream fragments separately. After PCR, check on the gel and make purifications for the two PCR fragments. In second round the top strand primer for the upstream fragment and the bottom strand primer for the downstream fragment should be used together with the two purified first-round PCR fragments. The extension reaction is started to join the overlapping region, followed by the regular PCR to amplify the joined upstream and downstream fragments containing mutated nucleotides at the seed motif of miRNA target tracks. Check on the gel after PCR, and make subclone first and sequencing to confirm the mutations. Then insert the mutated 3’-UTR fragment into your testing vector.

### Akt activity assay

To measure Akt kinase activities of in cells or tumor tissues, Akt activity assay was performed as previous described [41]. The immune complexes were then incubated with a biotinylated peptide substrate that became phosphorylated in the presence of activated Akt. The phosphorylated substrates, which reflected the activity of Akt kinase in the extract, was then quantified with the K-LISA Akt Activity Kit (Calbiochem, Darmstadt, Germany) that comprises a primary antibody recognizing the phosphorylated substrate peptides.

### Animal study

All mouse experiments were approved by The Institutional Animal Care and Use Committee of Sun Yat-sen University and the approval-No. was L102012016110D. The 6-week-old BALB/c-nu mice were randomly divided into four groups (*n* = 6 per group). The PC-3 cells (1 × 10^6^, 1 × 10^5^, 1 × 10^4^ and 1 × 10^3^) were inoculated subcutaneously together with Matrigel (final concentration of 25%) into the inguinal folds of the nude mice respectively. In the experiment testing, animals were injected with 100 μl agomir-133a-3p or agomir scramble through the lateral tail vein every four days for 4 weeks. The mice were sacrificed 35 days after inoculation and the tumors were excised and weighted. For the bone metastasis study, BALB/c-nu mice (5–6 weeks old) were anaesthetized and inoculated into the left cardiac ventricle with 1 × 10^5^ PC-3 cells in 100 μl of PBS. Agomir-133a-3p was injected through tail vein 2 days after inoculation of PC-3 cells. Osteolytic lesions were identified on radiographs as radiolucent lesions in the bone. The area of the osteolytic lesions was measured using the Metamorph image analysis system and software (Universal Imaging Corporation), and the total extent of bone destruction per animal was expressed in square millimeters. Each bone metastasis was scored based on the following criteria: 0, no metastasis; 1, bone lesion covering < 1/4 of the bone width; 2, bone lesion involving 1/4~ 1/2 of the bone width; 3, bone lesion across 1/2~ 3/4 of the bone width; and 4, bone lesion > 3/4 of the bone width. The bone metastasis score for each mouse was the sum of the scores of all bone lesions from four limbs.

### Flow cytometric analysis

Flow cytometric analyzed of apoptosis were used the FITC Annexin V Apoptosis Detection Kit I (BD, USA), and was presented as protocol described. Briefly, cells were dissociated with trypsin and resuspended at 1 × 10^6^ cells/mL in binding buffer with 50 μl/ml FITC Annexin V and 50 ul/ml PI. The cells were subsequently incubated for 15 min at room temperature, and then were analyzed by Gallios flow cytometer (Beckman Coulter, USA). The cell’s inner mitochondrial membrane potential (Δψm) was detected by flow cytometric using MitoScreen JC-1 staining kit (BD), and was presented as protocol described. Briefly, cells were dissociated with trypsin and resuspended at 1 × 10^6^ cells/mL in Assay Buffer, and then incubated at 37 °C for 15 min with 10 μl/ml JC-1. Before analyzed by flow cytometer, cells were washed twice by Assay Buffer. Flow cytometry data were analyzed using FlowJo 7.6 software (TreeStar Inc., USA).

### Caspase-9 or − 3 activity assays

Activity of caspase-9 or − 3 was analysis by spectrophotometry using Caspase-9 Colorimetric Assay Kit or Caspase-3 Colorimetric Assay Kit (Keygen, China), and was presented as protocol described. Briefly, 5 × 10^6^ cells or 100 mg fresh tumor tissues were washed with cold PBS and resuspended in Lysis Buffer and incubated on ice for 30 min. Mixed the 50 μl cell suspension, 50 μl Reaction Buffer, and 5 μl Caspase-3/− 9 substrate, and then incubated at 37 °C for 4 h. The absorbance was measured at 405 nm, and BCA protein quantitative analysis was used as the reference to normal each experiment groups.

### Side population analysis

The cell suspensions were labeled with Hoechst 33,342 (Molecular probes – #H-3570) dye for side population analysis as per standard protocol [42]. Briefly, cells were resuspended at 1× pre-warmed OptiMEM (Gibco, USA) containing 2% FBS (Gibco, USA) at a density of 106/mL. Hoechst 33,342 dye was added at a final concentration of 5 lg/mL in the presence or absence of verapamil (50 lmol/L; Sigma) and the cells were incubated at 37 °C for 90 min with intermittent shaking. At the end of the incubation, the cells were washed with OptiMem containing 2% FBS and centrifuged down at 4 °C, and resuspended in ice-cold OptiMem containing 2% FBS and 10 mmol/L HEPES. Propidium iodide (Sigma, USA) at a final concentration of 2 lg/mL was added to the cells to gate viable cells. The cells were filtered through a 40-lm cell strainer to obtain single cell suspension before sorting. Analysis and sorting was done on a FACS AriaI (Becton Dickinson). The Hoechst 33,342 dye was excited at 355 nm and its dual-wavelength emission at blue and red region was plotted to get the SP scatter.

### Spheroid formation assay

Cells (500 cells/well) were seeded into 6-well Ultra Low Cluster plates (Corning) and cultured in suspension in serum-free DMEM-F12 (BioWhittaker), supplemented with B27 (1:50, Invitrogen), 20 ng/ml endothelial growth factor (EGF; BD Biosciences), 0.4% bovine serum albumin (Sigma), and 4 mg/ml insulin (Sigma). After 10–12 days, the number of cell spheroids (tight, spherical, non-adherent masses > 50 μm in diameter) were counted, and images of the spheroids were scored under an inverse microscope (spheroids formation efficiency = colonies/input cells× 100%).

### High throughput data processing and visualization

The miRNAs expression levels and clinical profile of PCa dataset were downloaded from The Cancer Genome Atlas (TCGA: https://tcga-data.nci.nih.gov/tcga/). The log2 values of miRNAs in each sample were analyzed using Excel 2010 and GraphPad 5, as well as statistically analyze the miRNAs expression level of all PCa tissues using paired t-test or unpaired t-test. The expression levels of miRNAs in each sample were analyzed as previously described [43].

### Statistical analysis

All values are presented as the mean ± standard deviation (SD). Significant differences were determined using the GraphPad 5.0 software (USA). One-way ANOVA was used to determine statistical differences between multiple testing and the post hoc test after ANOVA is Tukey. Unpaired or paired t-test was used to determine statistical differences between two groups. The chi-square test was used to analyze the relationship between miR-133a-3p expression and clinicopathological characteristics. Survival curves were plotted using the Kaplan Meier method and compared by log-rank test. *P* < 0.05 was considered statistical significant. All experiments were repeated three times.

## Results

### miR-133a-3p expression is reduced in bone metastatic PCa tissues

**Fig. 1:**
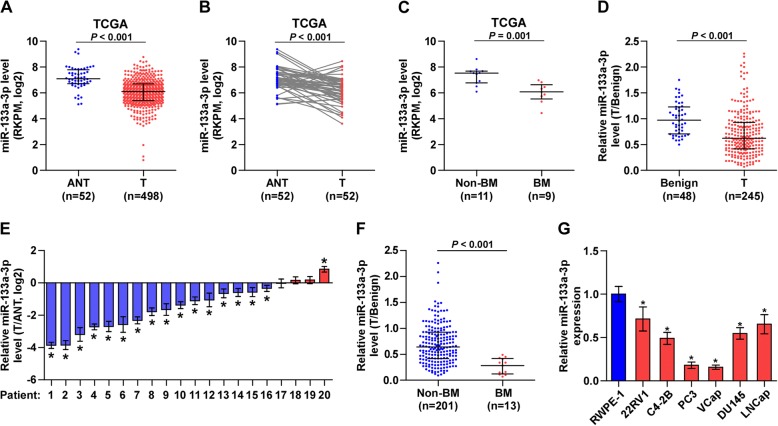
miR-133a-3p expression is reduced in PCa tissues and further downregulated in bone metastatic PCa tissues. **a** miR-133a-3p expression levels was decreased in PCa tissues compared with that in adjacent normal tissues (ANT) by analyzing the PCa miRNA sequencing dataset from TCGA (ANT, *n* = 52; PCa, *n* = 498). **b** miR-133a-3p expression levels was reduced in 52 paired PCa tissues compared with that in the matching ANT by analyzing the PCa miRNA sequencing dataset from TCGA. **c** miR-133a-3p expression levels was further decreased in the primary tumors of the patients with bone metastases (BM) compared with that in the primary tumors of the patients without bone metastases (non-BM) by analyzing the PCa miRNA sequencing dataset from TCGA. (non-BM, *n* = 11; BM, *n* = 9). **d** Real-time PCR analysis of miR-133a-3p expression in 48 benign prostate lesions tissues and 245 PCa tissues. Transcript levels were normalized to *U6* expression. Lines represent median and lower/upper quartiles. **e** Real-time PCR analysis of miR-133a-3p expression in 20 paired PCa tissues (miR-133a-3p expression level in PCa tissues: miR-133a-3p expression level in ANT). Transcript levels were normalized to *U6* expression. **f** Real-time PCR analysis of miR-133a-3p expression in 201 non-bone metastatic and 13 bone metastatic PCa samples. Transcript levels were normalized to *U6* expression. Lines represent median and lower/upper quartiles. *P < 0.05. **g** Real-time PCR analysis of miR-133a-3p expression levels in normal prostate epithelial cell (RWPE-1), primary PCa cell 22RV1, bone metastatic PCa cell lines (PC-3, C4-2B and VCaP) and brain metastatic cell line DU145 and lymph node metastatic cell line LNCaP. Transcript levels were normalized to *U6* expression. *P < 0.05

To determine the clinical significance of miR-133a-3p in PCa, we first analyzed several publicly available miRNA datasets of PCa from The Cancer Genome Atlas (TCGA) and ArrayExpress. As shown in Fig. 1a and b .and Additional file 6: Figure S1A-C, miR-133a-3p level was reduced in primary PCa tissues compared with that in the adjacent normal tissues (ANT) or benign prostate lesion tissues. Interestingly, we found that miR-133a-3p expression was further decreased in the primary tumors of the patients with bone metastasis (BM) compared with that in the primary tumors of the patients without bone metastases (Non-BM) (Fig. 1c). We further examined miR-133a-3p expression levels in our clinical PCa tissues and benign prostate lesions, including benign prostate hyperplasia and prostatitis, and found that miR-133a-3p expression was reduced in primary PCa tissues compared with that in benign prostate lesion tissues (Fig. 1d and e), and particularly in bone metastatic PCa tissues (Fig. 1f). We further examined the expression levels of miR-133a-3p in normal prostate epithelial cells RWPE-1 and other 6 PCa cells and found that miR-133a-3p expression were differentially downregulated compared with RWPE-1, especially in bone metastatic PCa cell lines (VCaP and PC-3) (Fig. 1g). Taken together, these results indicated that low expression of miR-133a-3p may be associated with the progression and bone metastasis of PCa.

### miR-133a-3p level negatively correlate with advanced clinicopathological characteristics and bone metastasis-free survival in PCa patients

**Fig. 2:**
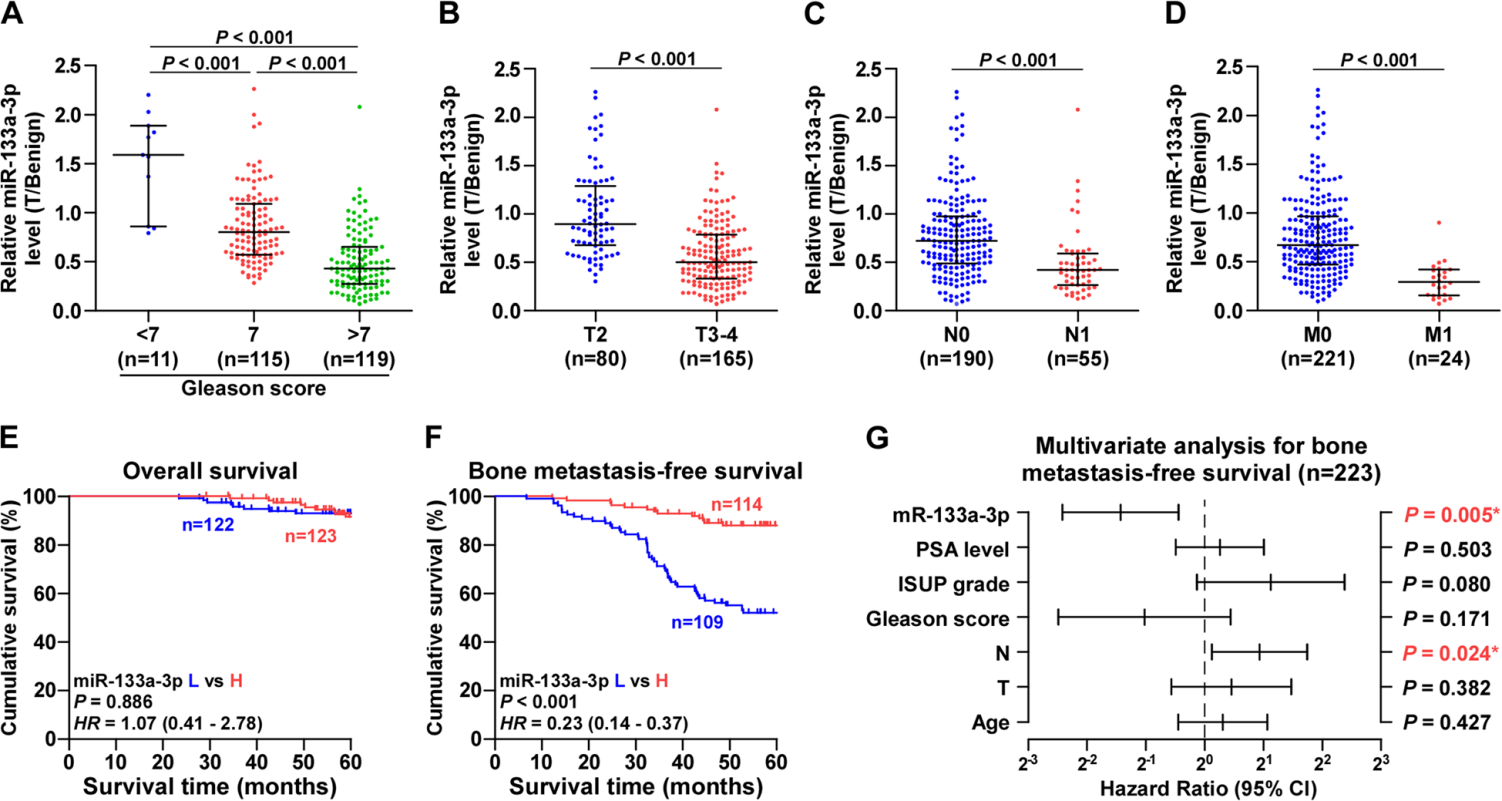
Low expression of miR-133a-3p correlates with poor clinicopathological characteristics and bone metastasis-free survival in PCa patients. **a** miR-133a-3p expression levels in PCa tissues with different Gleason score. **b** miR-133a-3p expression levels in PCa tissues with different tumor volume. **c** miR-133a-3p expression levels in PCa tissues with different lymph node metastasis status. **d** miR-133a-3p expression levels in PCa tissues with different distant metastasis status. **e** Kaplan–Meier analysis of overall survival curves of PCa patients with high miR-133a-3p expression (*n* = 123) versus low miR-133a-3p expression (*n* = 122). **f** Kaplan–Meier analysis of bone metastasis-free survival curves of PCa patients with high miR-133a-3p expression (*n* = 114) versus low miR-133a-3p expression (*n* = 109). **g** Multivariate Cox regression analysis to evaluate the significance of the association between miR-133a-3p expression and bone metastasis-free survival. HR values were presented by log2 transformation

The clinical correlation of miR-133a-3p expression levels with clinicopathological characteristics in PCa patients was further analyzed in our PCa tissues and PCa dataset from TCGA. As shown in Fig. 2a-d, Additional file 7: Table S6 and Additional file 8: Figure S2A-D, miR-133a-3p expression levels inversely correlated with Gleason grade, T classification, N classification and M classification in PCa patients. Kaplan-Meier survival analysis demonstrated that PCa patients with low miR-133a-3p expression showed shorter bone metastasis-free and progression-free survival, but had no effect on overall survival in PCa patients (Fig. 2e and f, Additional file 8: Figure S2E and F). Univariate Cox-regression analysis indicated patients with low miR-133a-3p expression had shorter bone metastasis-free survivals (*P* < 0.001; hazard ratio = 0.19, 95% CI = 0.11 to 0.36) compared to patients with high miR-133a-3p expression (Additional file 9: Table S7). Multivariate Cox regression analysis revealed that low expression of miR-133a-3p may be used as independent factors to predict bone metastasis-free survival (Fig. 2g and Additional file 10: Table S8). Collectively, our results indicated that low expression of miR-133a-3p strongly and positively with poor bone metastasis-free survival in PCa patients.

### Therapeutic effect of agomir-133a-3p on bone metastasis of PCa in vivo

**Fig. 3:**
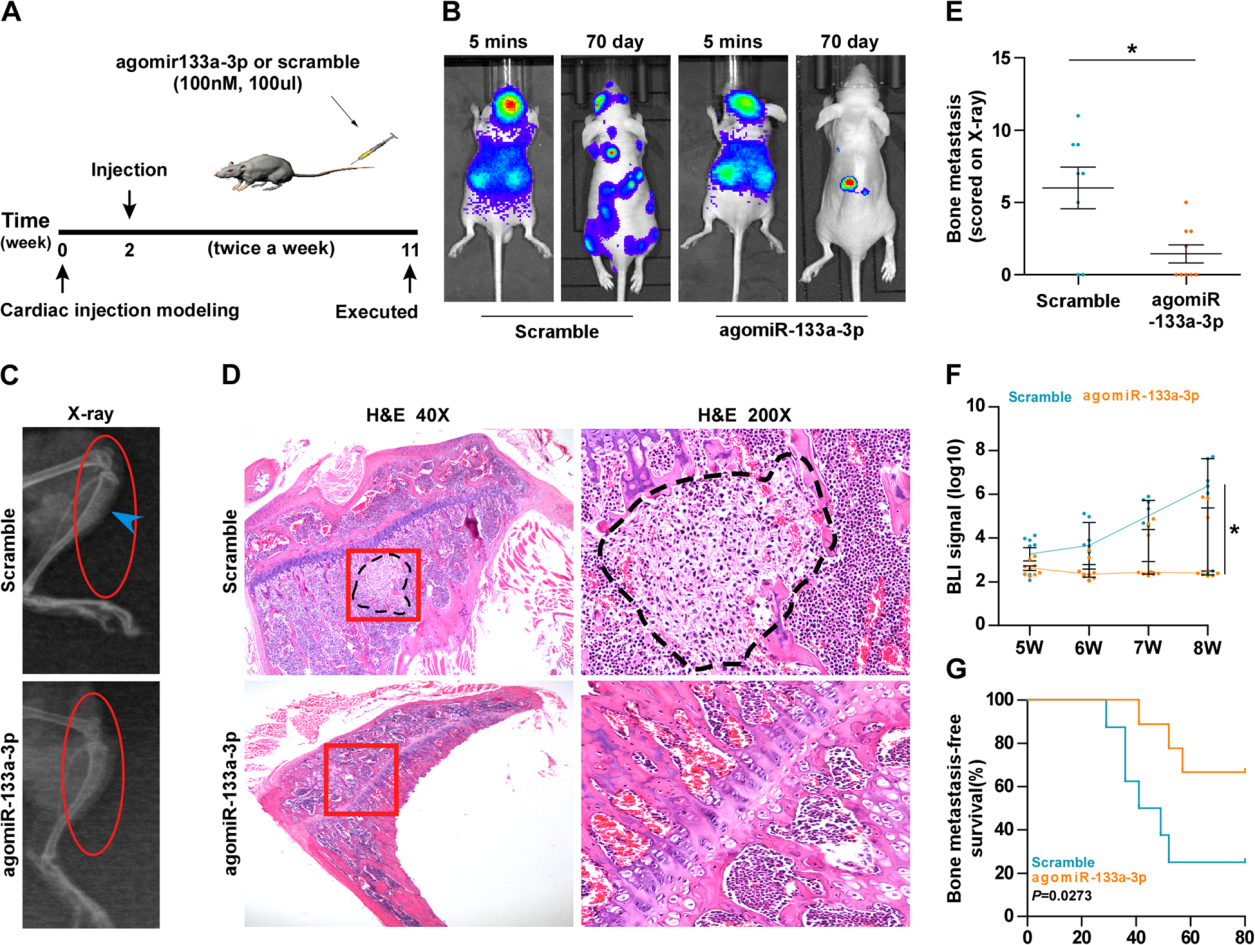
Upregulating miR-133a-3p represses bone metastasis of PC-3 cells in vivo. **a** Schematic model illustrating the time and route of agomir-133a-3p or scramble administration in a mouse model of bone metastasis. **b** Representative BLIs signal of bone metastasis of a mouse from the indicated groups of mice at 5 mins and 70 day respectively. **c** Representative radiographic images of bone metastases in the indicated mice (arrows indicate osteolytic lesions). **d** Representative H&E-stained sections of tibias from the indicated mouse. Scale bar, 500 μm (40× magnification) and 100 μm (200× magnification). **e** The sum of bone metastasis scores for each mouse in tumor-bearing mice inoculated with vector (*n* = 8) or agomir-133a-3p (*n* = 9) cells. **f** Quantification of the BLI signaling in the scramble or agomir-133a-3p groups at 5, 6, 7 and 8 weeks respectively. *P < 0.05. **g** Kaplan-Meier analysis of mouse bone metastasis-free survival in the scramble or agomir-133a-3p groups

To investigate the therapeutic effect of miR-133a-3p on the bone metastasis of PCa in vivo, a mouse intracardial model was used, where the luciferase-labeled vector PC-3 cells were inoculated into the left cardiac ventricle of male nude mice. The agomir-133a-3p or scramble was then injected through tail vein respective every four days for 6 weeks after inoculation of PC-3 cells (Fig. 3a). As shown in Fig. 3b and c, mice injected with agomir-133a-3p exhibited less bone metastasis ability compared with the control group by bioluminescence imaging (BLI) and X-ray. In addition, injection of agomir-133a-3p dramatically reduced the tumor burden in bone by H&E staining (Fig. 3d). Furthermore, injection of agomir-133a-3p decreased bone metastatic score and osteolytic area of tumors, and prolong bone metastasis-free survival compared to the control group (Fig. 3e-g). To circumvent the effects of agomir-133a-3p on other types of cells except PCa cells, we further constructed miR-133a-3p-stably expressing PCa cells via exogenously overexpressing miR-133a-3p via virus transduction in PC-3cells (Additional file 11: Figure S3A). Consistently, we found that upregulating miR-133a-3p repressed bone metastasis ability of PC-3 cells, decreased tumor burden, and extended bone metastasis-free survival compared to the control group (Additional file 11: Figure S3B-G). Collectively, our results demonstrate that upregulating miR-133a-3p represses the bone metastasis of PCa in vivo.

### Upregulation of miR-133a-3p inhibits cancer stem cell characteristics

**Fig. 4:**
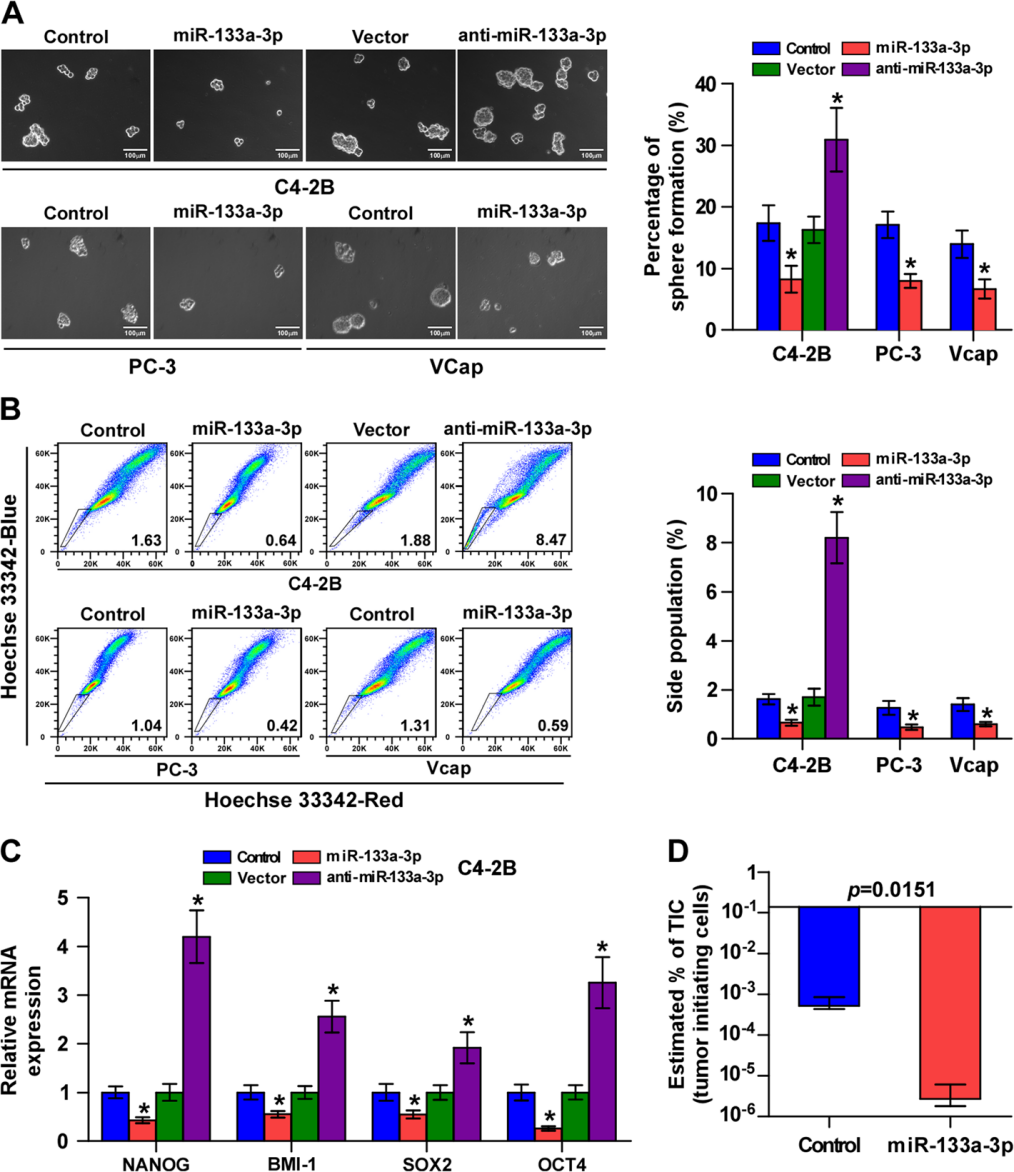
Upregulation of miR-133a-3p inhibits CSCs-like phenotypes in PCa cells. **a** Representative images of spheroids formed at 200-fold magnification were counted. Histograms showed the mean number of spheroids formed. Scale bars, 50 μm. Error bars represent the mean ± S.D. of three independent experiments. *P < 0.05. Scale bar, 100 μm. **b** Hoechst 33,342 dye exclusion assay showed that overexpressing miR-133a-3p reduced the fraction of side population, whereas silencing miR-133a-3p increased the fraction. *P < 0.05. **c** Real-time PCR analysis of OCT4A, SOX2, NANOG and BMI-1 expression. GAPDH was used as the loading control. Error bars represent the mean ± SD of three independent experiments. Error bars represent the mean ± S.D. of three independent experiments. *P < 0.05. **d** The estimated percentage of tumor initiating cells required in the indicated mice group

Accumulating studies have reported that cancer stem cells (CSCs) are the critical driver the critical driver of tumor progression and metastasis [44, 45]. Therefore, we further investigated the effects of miR-133a-3p on CSCs-like phenotypes in PCa cells via exogenously overexpressing miR-133a-3p via virus transduction in C4-2B and VCaP cells, and endogeneously silenced miR-133a-3p by transfecting anti-miR-133a-3p in C4-2B (Additional file 11: Figure S3A). Sphere formation assay was carried out and the results revealed that overexpression of miR-133a-3p reduced sphere formation ability in PCa cells, while silencing miR-133a-3p increased sphere formation ability (Fig. 4a). Side population (SP) analysis showed that upregulating of miR-133a-3p decreased, while silencing miR-133a-3p increased the fraction of SP cells (Fig. 4b). In addition, real-time PCR analysis demonstrated that upregulation of miR-133a-3p reduced, while silencing miR-133a-3p enhanced the mRNA expression levels of pluripotency-associated markers, including NANOG, BMI-1, OCT4 and SOX2 (Fig. 4c and Additional file 12: Figure S4A and B).

The effect of miR-133a-3p on the tumorigenesis of PCa cells was further investigated in vivo. As shown in Fig. 4d, the number of tumor initiating cells (TICs) required to develop tumor in mice were significantly reduced in the miR-133a-3p-overexpressing mice group. Furthermore, the tumors formed by miR-133a-3p-overexpressing cells were smaller than tumors in the control group after implantation of 1 × 10^6^, 1 × 10^5^ or 1 × 10^4^ cells (Additional file 12: Figure S4C). Importantly, the number of tumors formed in miR-133a-3p-overexpressing mice group was dramatically less than that in the control groups after inoculation of 1 × 10^5^ or 1 × 10^4^ (Additional file 12: Figure S4D). Collectively, these findings indicate that miR-133a-3p inhibits CSCs characteristics of PCa cells in vitro and in vivo.

### Upregulation of miR-133a-3p attenuates anoikis resistance in PCa cells

**Fig. 5:**
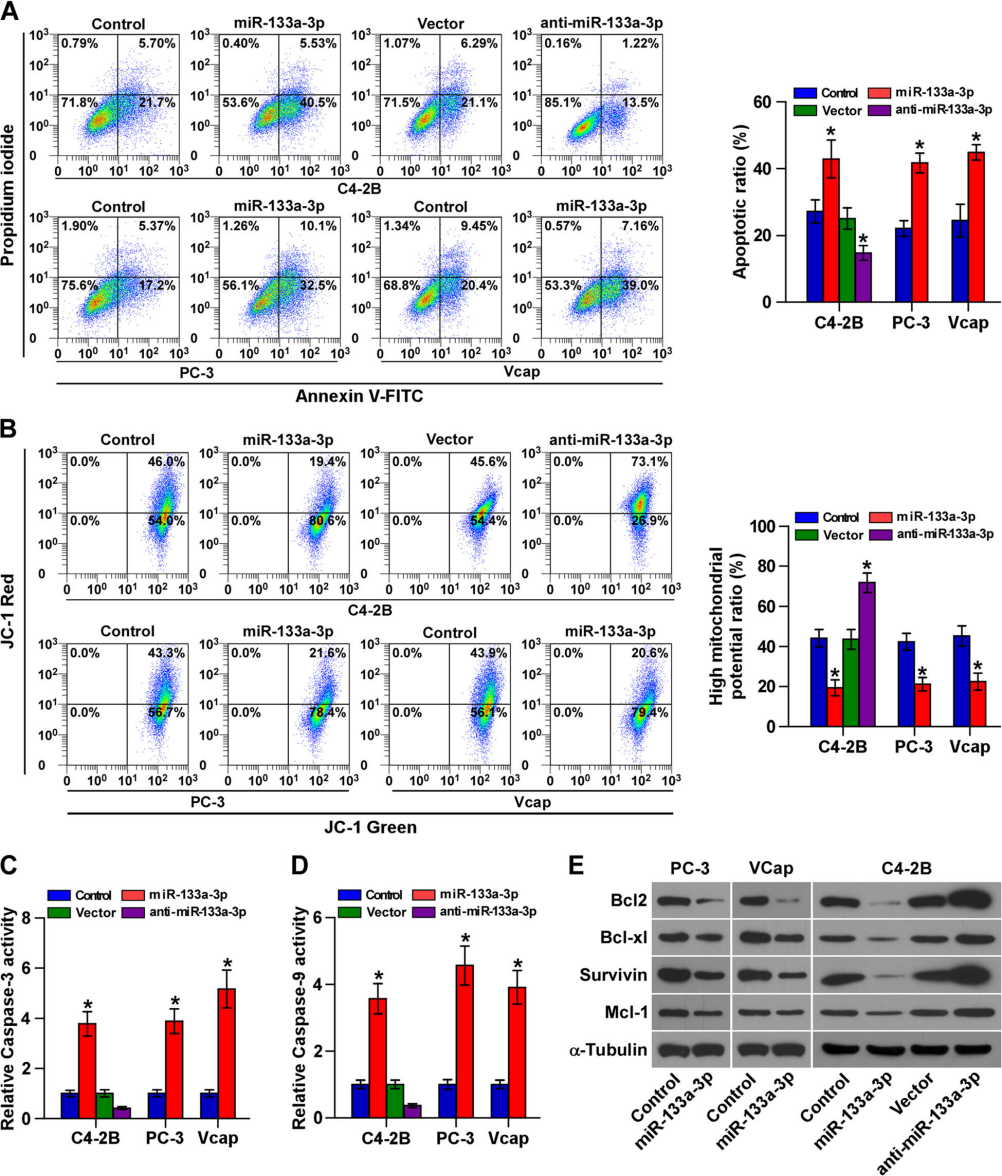
Upregulation of miR-133a-3p attenuates anoikis resistance in PCa cells. **a** Annexin V-FITC/PI staining of the indicated cells. Error bars represent the mean ± S.D. of three independent experiments. *P < 0.05. **b** The JC-1 staining in the indicated cells. Error bars represent the mean ± S.D. of three independent experiments. *P < 0.05. **c** and **d** Analysis of the activities of caspase-3 (**c**) and caspase-9 (**d**) were detected by the cleaved forms of these two proteins. Error bars represent the mean ± S.D. of three independent experiments. *P < 0.05. **e** Western blotting analysis of Bcl-2, Survivin, Mcl-1 and Bcl-xL in the indicated cells. α-Tubulin served as the loading control

Several lines of evidence have shown that TICs have the capacity to survive under suspension conditions, namely anoikis resistance, which is a major hallmark of metastasis in cancer [46, 47]. Therefore, we further examined the effects of miR-133a-3p on anoikis resistance in PCa cells. As shown in Fig. 5a, miR-133a-3p overexpression increased, while silencing miR-133a-3p decreased the apoptosis rate of PCa cells. Mitochondrial membrane potential assay was further performed and the results showed that miR-133a-3p overexpression reduced, while silencing miR-133a-3p increased, the mitochondrial potential of PCa cells (Fig. 5b). The effect of miR-133a-3p on the expression levels of the anti-apoptotic proteins, Bcl-2, Bcl-xL, Survivin and Mcl-1, and the activity of caspase-3 or − 9 were examined. As shown in Fig. 5c-e and Additional file 13: Figure S5A-C, upregulating miR-133a-3p increased, while silencing miR-133a-3p reduced the activity of caspase-3 or − 9 in PCa cells; conversely, upregulating miR-133a-3p decreased, while silencing miR-133a-3p enhanced the mRNA and proteins expression levels of these anti-apoptotic. Collectively, these results indicated that miR-133a-3p abrogates anoikis resistance in PCa cells.

### miR-133a-3p targets several cytokine receptors

**Fig. 6:**
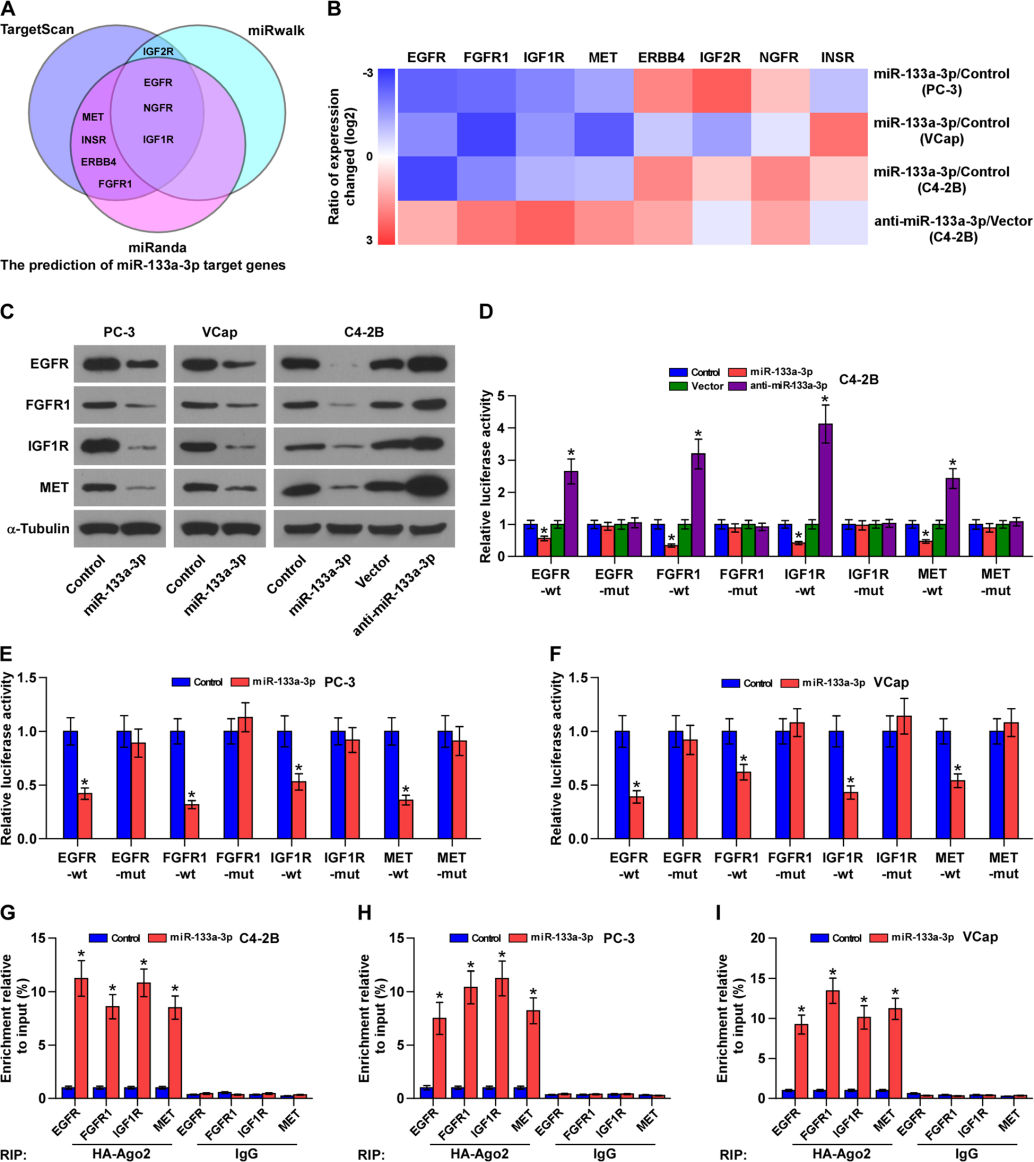
miR-133a-3p targets EGFR, FGFR1, IGF1R and MET. **a** Predicted target of miR-133a-3p in TargetScan, miRwalk and miRanda. **b** Real-time PCR analysis of EGFR, ERBB4, FGFR1, IGF1R, IFG2R, IN2R, MET and NGFR expression in the indicated PCa cells. Transcript levels were normalized by GAPDH expression. Error bars represent the mean ± s.d. of three independent experiments. *P < 0.05. **c** Western blotting of EGFR, FGFR1, IGF1R and MET expression in the indicated cells. α-Tubulin served as the loading control. **d-f** Luciferase assay of cells transfected with pmirGLO-3′UTR reporter in the indicated PCa cells, respectively. *P < 0.05. **g-i** MiRNP IP assay showing the association between miR-133a-3p and EGFR, FGFR1, IGF1R and MET transcripts in PCa cells. Pulldown of IgG antibody served as the negative control. *P < 0.05

By analyzing several available algorithms TargetScan, miRanda and miRWalk, we found that several cytokine receptors, including EGFR, ERBB4, FGFR1, IGF1R, IFG2R, IN2R, MET and NGFR, may be potential target of miR-133a-3p (Fig. 6a). Real-time PCR analysis showed that upregulating miR-133a-3p reduced, while silencing miR-133a-3p increased the mRNA expression levels of EGFR, FGFR1, IGF1R and MET, but not of ERBB4, IGF2R, NGFR and INSR (Fig. 6b). Western blotting revealed that upregulating miR-133a-3p reduced, while silencing miR-133a-3p increased EGFR, FGFR1, IGF1R and MET expression at the protein level (Fig. 6c). Luciferase assay revealed that upregulating miR-133a-3p decreased, while silencing miR-133a-3p increased the reporter activity of the 3′UTRs of EGFR, FGFR1, IGF1R and MET transcripts, but had no effect on mutant reporter activity (Fig. 6df-f and Additional file 14: Figure S6). RNA immunoprecipitation (IP) assay demonstrated a selective association of miR-133a-3p with EGFR, FGFR1, IGF1R and MET transcripts (Fig. 6gi-i). Consequently, our results reveal that EGFR, FGFR1, IGF1R and MET are the direct targets of miR-133a-3p in PCa cells.

### miR-133a-3p inhibits PI3K/AKT signaling pathway

**Fig. 7:**
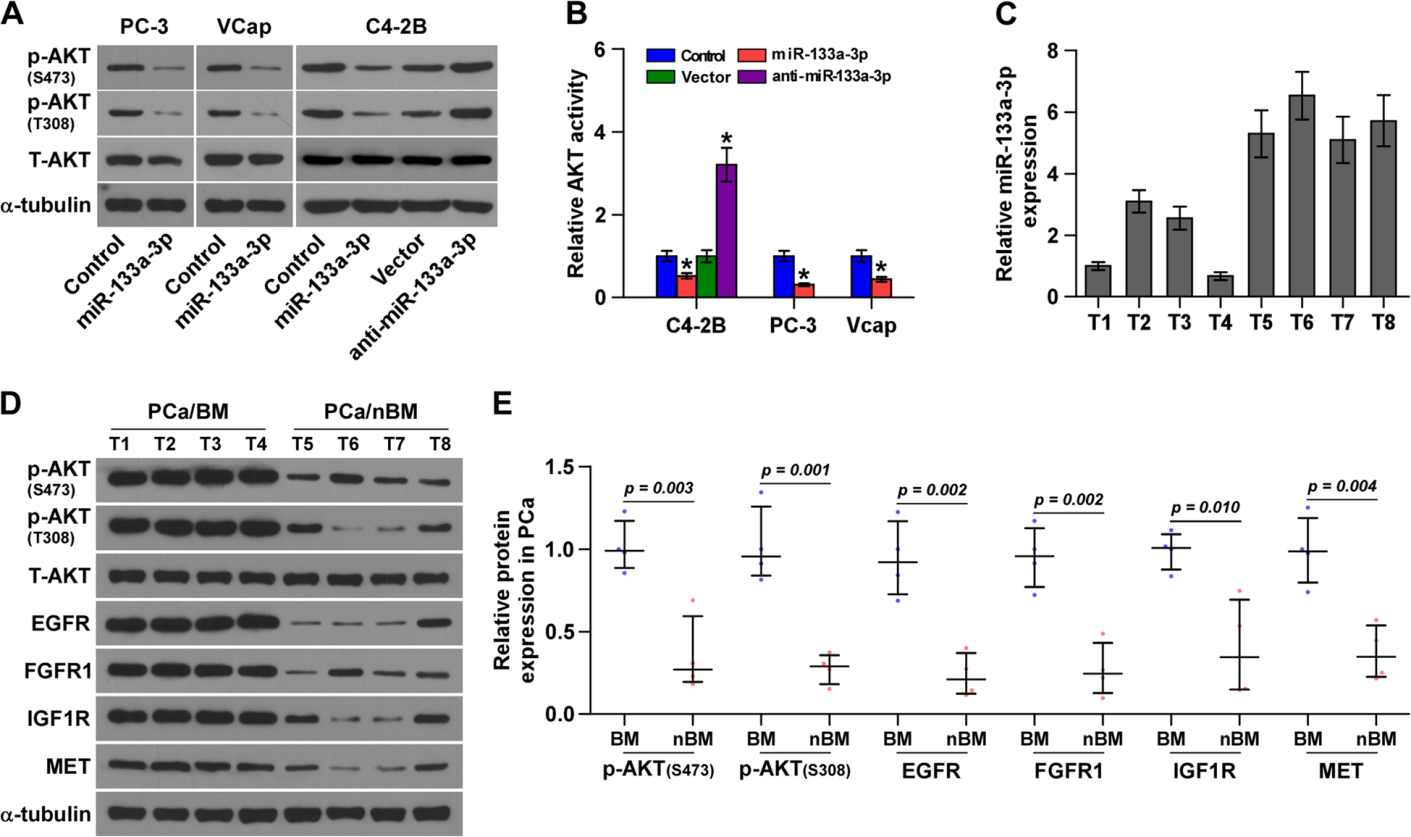
miR-133a-3p negatively regulates EGFR, FGFR1, IGF1R and MET expression, and PI3K/AKT signaling activity. **a** Western blotting of p-AKT expression in the indicated cells at S473 and T308. α-Tubulin served as the loading control. **b** Luciferase reporter analysis of AKT signaling activity in the indicated cells. *P < 0.05. **c** and **d** Analysis of miR-133a-3p expression with EGFR, FGFR1, IGF1R and MET, and p-AKT expression in 4 bone metastatic PCa tissues (T1–4) and 4 non-bone metastatic PCa tissues (T5–8). U6 was used as the control for RNA loading. miR-133a-3p expression levels were normalized to that miR-133a-3p expression of sample one. Each bar represents the mean ± SD of three independent experiments. *P < 0.05. α-tubulin was used as loading control. **e** Relative expression levels of EGFR, FGFR1, IGF1R and MET, and p-AKT at S473 and p-AKT at T308 expression in PCa tissues.The expression levels of EGFR, FGFR1, IGF1R and MET, and p-AKT expression were quantified by densitometry using Image J, and normalized to the levels of α-tubulin respectively. The sample with the lowest expression of each protein was used as a standard

Numerous literatures have reported that several cytokines, including EGF [7], IGF-1 [8], insulin [9] and CaM [10], has been reported to be a primary manner responsible for activation of PI3K/Akt pathway. And our results above demonstrated that miR133a-3p simultaneously targeted multiple cytokine receptors, including EGFR, FGFR1, IGF1R and MET, suggesting that miR133a-3p may have an influence on activity of PI3K/Akt signaling. Western blotting analysis showed that upregulation of miR-133a-3p decreased the phosphorylation levels of AKT, whereas silencing miR-133a-3p increased their expression (Fig. 7a). The analysis of AKT activity by luciferase reporter assays revealed that AKT activity was decreased in miR-133a-3p-overexpressing cells and increased in miR-133a-3p -silenced cells (Fig. 7b). Importantly, ELISA assays showed that upregulation or downregulation of miR-133a-3p had no significant effect on EGF, bFGF, IGF1, IGF2, NGF and HGF concentration in the supernantant of PCa cells (Additional file 15: Figure S7A-C). Taken together, these results indicated that miR-133a-3p negatively regulates AKT signaling activity via inhibiting cytokine receptors rather than cytokines expression, in PCa cells.

### AKT signaling is essential for the tumor-promoting role of anti-miR-133a-3p in PCa

To further determine whether AKT signaling is involved in the pro-tumor role of miR-133a-3p in PCa cells, we applied an allosteric AKT inhibitor MK-2206 to miR-133a-3p-silencing PCa cells. As shown in Additional file 16: Figure S8A and B, inhibition of AKT activity by MK-2206 decreased the sphere formation ability and mitochondrial membrane potential in miR-133a-3p-silencing PCa cells. Conversely, inhibition of AKT kinase activity abrogated the anti-apoptosis role of miR-133a-3p silencing in PCa cells (Additional file 16: Figure S8C). Taken together, these findings indicated that low expression of miR-133a-3p promotes the tumor progression and metastasis via activating the AKT signaling pathway in PCa.

### Clinical relation of miR-133a-3p with EGFR, FGFR1, IGF1R and MET expression, and PI3K/AKT signaling activity in human PCa tissues

To determine the clinical correlation of miR-133a-3p with EGFR, FGFR1, IGF1R and MET expression, and PI3K/AKT signaling activity in clinical PCa tissues, the miR-133a-3p expression and protein levels of EGFR, FGFR1, IGF1R and MET and pAKT expression were examined in four bone metastatic and four non-bone metastatic PCa tissues. As shown in Fig. 7c-e, miR-133a-3p expression levels were downregulated in bone metastatic PCa tissues (T1–4) compared with that in non-bone metastatic PCa tissues (T5–8). By contrast, protein expression of EGFR, FGFR1, IGF1R, MET and pAKT in bone metastatic PCa tissues was increased compared with that in non-bone metastatic PCa tissues. Taken together, our results indicate that miR-133a-3p inhibits bone metastasis of PCa via inactivating PI3K/AKT signaling by simultaneously targeting EGFR, FGFR1, IGF1R and MET.

## Discussion

Numerous studies have demonstrated that copy number variation, chromosomal rearrangements and genetic mutations are implicated in the progression and metastasis of PCa [48–50]. In metastatic PCa tumor tissues, phosphatase and tensin homolog (PTEN) loss-of-function mutations or genomic alterations in components of the PI3K/AKT signaling even reaches up to 70% [48, 51], supporting the critical roles of PI3K/AKT signaling in metastatic PCa. Furthermore, epigenetic regulations are emerging as important contributing factors for the unrestrained activation of AKT signaling [52]. Among these factors, aberrant expression of miRNAs constitutes a compelling component in epigenome. miR-27b has been reported to decreased in diffuse large B-cell lymphoma (DLBCL). Forced expression of miR-27b suppressed DLBCL cell proliferation and tumor growth via repressing PI3K/AKT pathway by targeting MET [53]. In addition, miR-508 directly targeted multiple phosphatases, including INPP4A, INPP5J and PTEN, resulting in constitutive activation of PI3K/Akt signaling, which promoted the aggressive phenotype of oesophageal squamous cell carcinoma [41]. In PCa, several miRNAs, including miR-16, miR-106b, miR-148a, miR-4534 and miR-195, have been reported to be implication in the activation of the PI3K/Akt signaling pathway [54–56]. In the current study, our results demonstrated that several cytokine receptors of Akt signaling, including EGFR, FGFR1, IGF1R and MET, were direct targets of miR-133a-3p in PCa cells. Downregulation of miR-133a-3p dramatically augmented the activity of PI3K/Akt signaling in PCa cells. Therefore, our results uncover a novel mechanism for the constitutive activation of PI3K/Akt signaling in bone metastasis of PCa.

The PI3K/Akt signaling cascade can be activated by several stimuli, including integrins, receptor tyrosine kinases, cytokine receptors and G-protein-coupled receptors, all of which can induce production of phospha- tidylinositol (3,4,5) trisphosphates (PIP3) by phosphoinositide 3-kinase (PI3K) [5, 6]. Among these, cytokine activation of PI3K/Akt pathway has been regarded as a primary manner mediating PI3K/Akt signaling cascade. In this manner, activation of PI3K/Akt signaling starts with the binding of cytokine to the corresponding receptor, such as EGF [7], IGF-1 [8] and insulin [9]. Excessive secretion of cytokines in an autocrine or paracrine manner, or increased expression of the corresponding receptors has been reported to contribute to constitutive activation of PI3K/AKT signaling [57–60]. However, how these cytokines and the receptors are simultaneously disrupted in cancers, resulting in the activation of PI3K/AKT signaling, remains unclear. In this study, through analyzing publicly available algorithms, we found that several cytokine receptors, including EGFR, ERBB4, FGFR1, IGF1R, IFG2R, IN2R, MET and NGFR, may be potential target of miR-133a-3p, in which only EGFR, FGFR1, IGF1R and MET were targeted by miR-133a-3p in PCa cells. Importantly, autocrine levels of the corresponding cytokines were not affected by upregulating or silencing miR-133a-3p in PCa cells. Taken together, our results suggest that miR-133a-3p represses AKT signaling activity via inhibiting cytokine receptors, but has no any effect on cytokines secretion in PCa cells.

Downexpression of miR-133a-3p has been extensively reported in a various types of cancer and predicted a poor prognosis [61–67]. However, several lines of evidence have shown that miR-133a-3p was upregulated in hepatocellular carcinoma [29], multiple myeloma [31], breast cancer [68] and osteosarcoma [69], suggesting that miR-133a-3p plays an oncogenic or tumor-suppressive miRNA depending on tumor types. Furthermore, low expression of miR-133a-3p has been correlated with the recurrence and distant metastasis of PCa [32–36]. However, the clinical significance of miR-133a-3p in the progression and bone metastasis of PCa, as well as the biological role of miR-133a-3p and its molecular mechanisms underlying bone metastasis of PCa have not been elucidated. In this study, our results demonstrated that miR-133a-3p was downregulated in PCa tissues and further reduced in bone metastatic PCa tissues. Low expression of miR-133a-3p closely correlated with advanced clinicopathological characteristics and poor bone metastasis-free survival in PCa patients. Furthermore, our results clarified that miR-133a-3p repressed bone metastasis of PCa via inactivating PI3K/AKT signaling by directly targeting EGFR, FGFR1, IGF1R and MET. Therefore, our results indicate that miR-133a-3p functions as a tumor-suppressive miRNA via disrupting PI3K/AKT signaling in bone metastasis of PCa.

Strikingly, our results revealed that the low miR-133-3p expression did not affect overall survival in PCa patients, although the low miR-133-3p levels positively and significantly correlated with advanced clinicopathological characteristics of PCa patients, including serum PSA levels, Gleason score and TNM status. In fact, these pathological parameters predict poor overall survival in PCa patients [70–74]. The possibility that low levels of miR-133a-3p were significantly associated with serum PSA levels, Gleason score and TNM status in PCa patients, but had no effect on overall survival in PCa patients is that the majority of our PCa samples were obtained from 2011 to 2016, and PCa patients have a relatively high rate of 5-year overall survival. Therefore, the solid conclusion about the prognostic prediction of miR-133a-3p expression levels in overall survival in PCa patients is well warranted to follow up in the following couples of years.

Circulating microRNAs have drawn a great deal of interest as promising novel non-invasive biomarkers for the early diagnosis of cancer. Recently, the involvement of circulating miR-133a-3p as a potential biomarker for diagnosis and prognosis of cancer are becoming increasingly appreciated. In breast cancer, higher miR-133a-3p levels in the plasma or serum of breast cancer patients provided considerable discrimination compared with the healthy controls [68, 75, 76]. Conversely, low expression level of miR-133a-3p expression was observer in the serum of astrocytomas and colorectal cancer patients with a high sensitivity and specificity [77, 78]. However, little is known about the potential applicable values of circulating miR-133a-3p in PCa and its bone metastatic phenotypes. In this study, our results demonstrated that low expression of miR-133a-3p strongly correlated with bone metastasis-free survival in PCa patients, suggesting that miR-133a-3p may serve as a potential bone metastasis diagnostic marker in PCa patients. However, whether miR-133a-3p expression in the serum or plasma samples of PCa patients may be used as a potential non-invasive marker to predict bone metastasis of PCa requires further investigation.

## Conclusion

In summary, our results demonstrate that miR-133a-3p inhibits bone metastasis of PCa by targeting multiple cytokine receptors, including EGFR, FGFR1, IGF1R and MET, leading to inactivation of PI3K/AKT signaling pathway. Thus, clearly clarifying the functional role and the underlying mechanism of miR-133a-3p in the bone metastasis of PCa will facilitates the development of novel anti-bone metastatic therapeutic methods against PCa.

## Additional files

Additional file 1: **Table S1.** A list of primers used in the reactions for clone PCR. (PDF 49 kb)

Additional file 2: **Table S2.** A list of primers used in the reactions for real-time RT-PCR. (PDF 60 kb)

Additional file 3: **Table S3.** The basic information of 20 paired prostate adenocarcinoma patients for miR-133a-3p expression analysis. (PDF 54 kb)

Additional file 4: **Table S4.** The basic information of 48 patients with benign prostate lesions for miR-133a-3p expression analysis. (PDF 47 kb)

Additional file 5: **Table S5.** The basic information of 245 prostate adenocarcinoma patients for miR-133a-3p expression analysis. (PDF 60 kb)

Additional file 6: **Figure S1.** miR-133a-3p is downregulated in PCa tissues. (A) miR-133a-3p expression levels was decreased in 26 paired PCa tissues compared with that in the matching ANT by analyzing the miRNA sequencing dataset of PCa from GSE76260. (B) miR-133a-3p expression levels was decreased in 32 individual PCa tissues compared with that in 32 ANT by analyzing the miRNA sequencing dataset of PCa from GSE76260. (C) miR-133a-3p expression levels was decreased in PCa tissues compared with that in benign prostate lesion tissues by analyzing the miRNA sequencing dataset of PCa from GSE36802 (Benign, *n* = 21; PCa, *n* = 21). (PDF 91 kb)

Additional file 7: **Table S6.** The relationship between miR-133a-3p expression level and clinical pathological characteristics in 245 patients with prostate adenocarcinoma. (PDF 61 kb)

Additional file 8: **Figure S2.** Low expression of miR-133a-3p correlates with poor clinicopathological characteristics and progression-free survival in PCa patients. (A) miR-133a-3p expression levels in PCa tissues with different Gleason score as assessed by TCGA. (B) miR-133a-3p expression levels in PCa tissues with different tumor volume as assessed by TCGA. (C) miR-133a-3p expression levels in PCa tissues with different lymph node metastasis status as assessed by TCGA. (D) miR-133a-3p expression levels in PCa tissues with different distant metastasis status as assessed by TCGA. (E) Kaplan–Meier analysis of overall survival curves of PCa patients with high miR-133a-3p expression (*n* = 247) versus low miR-133a-3p expression (n = 247) as assessed by TCGA. (F) Kaplan–Meier analysis of progression-free survival curves of PCa patients with high miR-133a-3p expression (*n* = 228) versus low miR-133a-3p expression (*n* = 219) as assessed by TCGA. (PDF 247 kb)

Additional file 9: **Table S7.** Univariate and multivariate analysis of factors associated with overall survival in 245 patients with prostate adenocarcinoma. (PDF 10 kb)

Additional file 10: **Table S8.** Univariate and multivariate analysis of factors associated with bone metastasis free survival in 223 patients with prostate adenocarcinoma. (PDF 10 kb)

Additional file 11: **Figure S3.** Real-time PCR analysis of miR-133a-3p expression in the indicated PC-3, C4-2B and VCaP cells. Transcript levels were normalized by U6 expression. Error bars represent the mean ± s.d. of three independent experiments. *P < 0.05. (PDF 173 kb)

Additional file 12: **Figure S4.** (A and B) Real-time PCR of NANOG, BMI-1, SOX2 and OCT4 in the indicated cells. Transcript levels were normalized by U6 expression. Error bars represent the mean ± s.d. of three independent experiments. *P < 0.05. (C) Histograms show the mean tumor weights of each group. *P < 0.05. (D) The number of tumor formation initiated by different amounts of PC-3 cells in nude mice. *n* = 6 per group. (PDF 104 kb)

Additional file 13: **Figure S5.** (A-C) Real-time PCR of BCL2, BCL-xL, Survivin and Mcl-1 in the indicated cells. Transcript levels were normalized by U6 expression. Error bars represent the mean ± s.d. of three independent experiments. *P < 0.05. (PDF 72 kb)

Additional file 14: **Figure S6.** Predicted miR-133a-3p targeting sequence and mutant sequences in 3′UTRs of EGFR, FGFR1, IGF1R and MET. (PDF 109 kb)

Additional file 15: **Figure S7.** (A-C) ELISA analysis of EGF, bFGF, IGF1, IGF2, NGF and HGF concentration in the supernatant of the indicated cells. (PDF 68 kb)

Additional file 16: **Figure S8.** AKT signaling was essential for the pro-tumor roles of silencing miR-133a-3p in PCa cells. (A) AKT inhibitors MK2206 (1 μM) decreased sphere formation ability in miR-133a-3p-silencing PCa cells.*P < 0.05. (B) AKT inhibitors MK2206 (1 μM) decreased the mitochondrial potential in miR-133a-3p-silencing PCa cells.*P < 0.05. (C) AKT inhibitors MK2206 (1 μM) promoted the apoptosis rate in miR-133a-3p-silencing PCa cells.*P < 0.05. (PDF 97 kb)

## Abbreviations

ANT: Adjacent normal tissues
Bcl-xl: BCL2 Like 1
BM: Bone metastasis
BMI1: Proto-oncogene polycomb ring finger
CSCs: Cancer stem cells
EGFR: Epidermal Growth Factor Receptor
ERBB4: Erb-B2 Receptor Tyrosine Kinase 4
FGFR1: Fibroblast Growth Factor Receptor 1
H&E: Hematoxylin and Eosin Stain
IGF1R: Insulin Like Growth Factor 1 Receptor
IGF2R: Insulin Like Growth Factor 2 Receptor
INSR: Insulin Receptor
Mcl-1: Myeloid Cell Leukemia Sequence 1
MET: MET Proto-Oncogene, Receptor Tyrosine Kinase
NANOG: Nanog homeobox
NGFR: Nerve Growth Factor Recepto
Non-BM: Non bone metastasis
OCT4A: POU class 5 homeobox 1A
PCR: Polymerase Chain Reaction
SOX2: SRY-Box 2
SP: Side population
Survivin: Baculoviral IAP Repeat Containing 5
TCGA: The Cancer Genome Atlas
TICs: Tumor initiating cells
