# Supplementary material for: Downregulation of miR-133a-3p promotes prostate cancer bone metastasis via activating PI3K/AKT signaling
Source: J Exp Clin Cancer Res. 2018 Jul 18;37:160. doi: 10.1186/s13046-018-0813-4 (PMC6052526; doi:10.1186/s13046-018-0813-4)
Supplement: Supplementary file 1 — Table S1. A list of primers used in the reactions for clone PCR. (PDF 49 kb) [file 13046_2018_813_MOESM1_ESM.pdf]

Table S1. A list of primers used in the reactions for clone PCR.

Gene	Sequence (5` – 3`)
miR-133a-clone-F	TCTTTAACCATTCTAGCTTTTCCAGG
miR-133a-clone-R	AAGCAAAGATGTTGAAAACAAAGACG
EGFR-3`UTR-1-F	CCACGGAGGATAGTATGAGCCC
EGFR-3`UTR-506-R	AGTGGAAGCCTTGAAGCAGAAC
FGFR1-3`UTR-24-F	GACTCCACCGTCAGCTGTAAC
FGFR1-3`UTR-565-R	TTCGCCTCACCATCCTCTG
IGF1R-3`UTR-2814-F	AGCTTGCCTTTTTCTGAGATGTCC
IGF1R-3`UTR-3235-R	GCTGCACCTTTGTGCTCAATG
MET-3`UTR-54-F	TGCCTGACCTTTAAAAGGCCATC
MET-3`UTR-554-R	TGGCAACAGAGTGAGATCCTGTC
