# Supplementary material for: Downregulation of miR-133a-3p promotes prostate cancer bone metastasis via activating PI3K/AKT signaling
Source: J Exp Clin Cancer Res. 2018 Jul 18;37:160. doi: 10.1186/s13046-018-0813-4 (PMC6052526; doi:10.1186/s13046-018-0813-4)
Supplement: Supplementary file 2 — Table S2. A list of primers used in the reactions for real-time RT-PCR. (PDF 60 kb) [file 13046_2018_813_MOESM2_ESM.pdf]

Table S2. A list of primers used in the reactions for real-time RT-PCR.

Gene	Sequence (5`– 3`)	product size (bp)
NANOG-F	TCCAACATCCTGAACCTCAGCTA	186
NANOG-R	AGTCGGGTTCACCAGGCATC	
BMI-1-F	TCGTTGTTCGATGCATTTCT	90
BMI-1-R	CTTTCATTGTCTTTTCCGCC	
SOX2-F	GTGAGCGCCCTGCAGTACAA	82
SOX2-R	GCGAGTAGGACATGCTGTAGGTG	
OCT4-F	TGAGTAGTCCCTTCGCAAGC	98
OCT4-R	GAGAAGGCGAAATCCGAAG	
EGFR-F	GTGACCGTTTGGGAGTTGATGA	104
EGFR-R	GGCTGAGGGAGGCGTTCTC	
FGFR1-F	CCTCTATGTGGGCATGGTTT	128
FGFR1-R	TACAGGAAGGACGATCTGGG	
IGF1R-F	AGTTATCTCCGGTCTCTGAGG	138
IGF1R-R	TCTGTGGACGAACTTATTGGC	
MET-F	TTCTGACCGAGGGAATCATCA	82
MET-R	CCTTCACTTCGCAGGCAGAT	
ERBB4-F	TGCCCTACAGAGCCCCAACTA	105
ERBB4-R	GCTTGCGTAGGGTGCCATTAC	
IGF2R-F	ACCTCTGACAAGACCAAGTA	82
IGF2R-R	GTCTTATCCTTTCCGCACTT	
NGFR-F	CCTGTCTATTGCTCCATCCTG	126
NGFR-R	GGGCGTCTGGTTCACTG	
INSR-F	ACTACTCCTTCTATGCCTTG	128
INSR-R	GATTTCTGACAAGCAGAGTT	
BCL2-F	GGTGGGGTCATGTGTGTGG	89
BCL2-R	CGGTTCAGGTACTCAGTCATCC	
BCL-XL-F	CTGCTGCATTGTTCCCATAG	289
BCL-XL-R	TTCAGTGACCTGACATCCCA	
survivin-F	TCCGGTTGCGCTTTCCT	121
survivin-R	TCTTCTTATTGTTGGTTTCCTTTGC	
MCL-1-F	GGACATCAAAAACGAAGACG	154
MCL-1-R	GCAGCTTTCTTGGTTTATGG	
GAPDH-F	TCCTCTGACTTCAACAGCGACAC	126
GAPDH-R	CACCCTGTTGCTGTAGCCAAATTC	
