# Supplementary material for: Downregulation of miR-133a-3p promotes prostate cancer bone metastasis via activating PI3K/AKT signaling
Source: J Exp Clin Cancer Res. 2018 Jul 18;37:160. doi: 10.1186/s13046-018-0813-4 (PMC6052526; doi:10.1186/s13046-018-0813-4)
Supplement: Supplementary file 3 — Table S3. The basic information of 20 paired prostate adenocarcinoma patients for miR-133a-3p expression analysis. (PDF 54 kb) [file 13046_2018_813_MOESM3_ESM.pdf]

Table S3. The basic information of 20 paired prostate adenocarcinoma patients for miR-133a-3p expression analysis.

		Cases (n)	Percentage (%)
Histologic	Acinar Type	20	100.0
	Other	0	0.0
Age	<62	9	45.0
	≥62	11	55.0
T classification	T1	0	0.0
	T2	6	30.0
	T3	14	70.0
	T4	0	0.0
N classification	N0	15	75.0
	N1	5	25.0
M classification	M0	19	95.0
	M1	1	5.0
Gleason score	≤6	1	5.0
	7	10	50.0
	≥8	9	45.0
ISUP Grade	1	1	5.0
	2	6	30.0
	3	3	15.0
	4	3	15.0
	5	7	35.0
Bone metastasis	Positive	1	5.0
status	Negative	19	95.0

* ISUP: International Society of Urological Pathology.
