# Supplementary material for: Downregulation of miR-133a-3p promotes prostate cancer bone metastasis via activating PI3K/AKT signaling
Source: J Exp Clin Cancer Res. 2018 Jul 18;37:160. doi: 10.1186/s13046-018-0813-4 (PMC6052526; doi:10.1186/s13046-018-0813-4)
Supplement: Supplementary file 4 — Table S4. The basic information of 48 patients with benign prostate lesions for miR-133a-3p expression analysis. (PDF 47 kb) [file 13046_2018_813_MOESM4_ESM.pdf]

Table S4. The basic information of 48 patients with benign prostate lesions for miR-133a-3p expression analysis.

		Cases (n)	Percentage (%)
Age	<62	26	54.2
	≥62	22	45.8
Type of diseases	Hyperplasia	30	62.5
	Prostatitis	18	37.5

* NA: Not available.
