# Supplementary material for: Downregulation of miR-133a-3p promotes prostate cancer bone metastasis via activating PI3K/AKT signaling
Source: J Exp Clin Cancer Res. 2018 Jul 18;37:160. doi: 10.1186/s13046-018-0813-4 (PMC6052526; doi:10.1186/s13046-018-0813-4)
Supplement: Supplementary file 5 — Table S5. The basic information of 245 prostate adenocarcinoma patients for miR-133a-3p expression analysis. (PDF 60 kb) [file 13046_2018_813_MOESM5_ESM.pdf]

Table S5. The basic information of 245 prostate adenocarcinoma patients for miR-133a-3p expression analysis.

		Cases (n)	Percentage (%)
Histologic	Acinar Type	241	98.4
	Other	4	1.6
Age	<62	122	49.8
	≥62	123	50.2
T classification	T1	0	0.0
	T2	80	32.7
	T3	160	65.3
	T4	5	2.0
N classification	N0	190	77.6
	N1	55	22.4
M classification	M0	221	90.2
	M1	24	9.8
Gleason score	≤6	11	4.5
	7	115	46.9
	≥8	119	48.6
ISUP Grade	1	11	4.5
	2	73	29.8
	3	42	17.1
	4	30	12.2
	5	89	36.3
PSA level	≤ 20ng/ml	201	82.0
	>20 ng/ml	17	6.9
Bone scan or CT or MRI results	Normal	13	5.3
	Abnormal	5	2.0
	Bone metastasis	9	3.7

	Equivocal	169	69.0
	N/A	76	31.0
The survival follow-up	Alive	228	93.1
	Death	17	6.9
The bone metastasis follow-up (exclude: BM at first)	Negative	159	64.9
	Positive	64	26.1
	N/A	22	9.0

* ISUP: International Society of Urological Pathology, N/A: Not available, PSA: Prostate-specific antigen.

** Other histologic cancers included: Duct adenocarcinoma, Squamous cell carcinoma.
