# Supplementary material for: Downregulation of miR-133a-3p promotes prostate cancer bone metastasis via activating PI3K/AKT signaling
Source: J Exp Clin Cancer Res. 2018 Jul 18;37:160. doi: 10.1186/s13046-018-0813-4 (PMC6052526; doi:10.1186/s13046-018-0813-4)
Supplement: Supplementary file 6 — Figure S1. miR-133a-3p is downregulated in PCa tissues. (A) miR-133a-3p expression levels was decreased in 26 paired PCa tissues compared with that in the matching ANT by analyzing the miRNA sequencing dataset of PCa from GSE76260. (B) miR-133a-3p expression levels was decreased in 32 individual PCa tissues compared with that in 32 ANT by analyzing the miRNA sequencing dataset of PCa from GSE76260. (C) miR-133a-3p expression levels was decreased in PCa tissues compared with that in benign prostate lesion tissues by analyzing the miRNA sequencing dataset of PCa from GSE36802 (Benign, n = 21; PCa, n = 21). (PDF 91 kb) [file 13046_2018_813_MOESM6_ESM.pdf]

Table S6. The relationship between miR-133a-3p expression level and clinical pathological characteristics in 245 patients with prostate adenocarcinoma.

Parameters	Number of cases	PLPP4 IHC expression		P values
		Low	High	
Histologic				
Acinar Type	241	119	122	0.370
Other	4	3	1	
Age				
<62	122	54	68	0.085
≥62	123	68	55	
T classification				
T1 – T2	80	18	62	<0.001*
T3 – T4	165	104	61	
N classification				
N0	190	77	113	<0.001*
N1	55	45	10	
M classification				
M0	221	99	122	<0.001*
M1	24	23	1	
Gleason score				
≤7	126	36	90	<0.001*
>7	119	86	33	
ISUP Grade				
≤3	126	36	90	<0.001*
>3	119	86	33	
PSA level				
≤20 ng/ml	169	74	95	0.005*
>20 ng/ml	76	48	28	

Bone scan or CT or MRI results				
Normal	201	96	105	<0.001*
Bone metastasis	13	13	0	

* ISUP: International Society of Urological Pathology, N/A: Not available, PSA: Prostate-specific antigen.
