# Supplementary material for: Downregulation of miR-133a-3p promotes prostate cancer bone metastasis via activating PI3K/AKT signaling
Source: J Exp Clin Cancer Res. 2018 Jul 18;37:160. doi: 10.1186/s13046-018-0813-4 (PMC6052526; doi:10.1186/s13046-018-0813-4)
Supplement: Supplementary file 7 — Table S6. The relationship between miR-133a-3p expression level and clinical pathological characteristics in 245 patients with prostate adenocarcinoma. (PDF 61 kb) [file 13046_2018_813_MOESM7_ESM.pdf]

Table S7. Univariate and multivariate analysis of factors associated with overall survival in 245 patients with prostate adenocarcinoma.

Characteristics	Univariate analysis		Multivariate analysis	
	HR (95% CI)	*P* values	HR (95% CI)	*P* values
Age (> 62)	19.18 (2.54-144.76)	0.004*	22.57 (2.92-174.22)	0.003*
T classification	1.84 (0.67-5.07)	0.240	1.86 (0.64-5.39)	0.254
N classification	0.78 (0.23-2.72)	0.700	0.80 (0.19-3.44)	0.769
M classification	4.28 (1.51-12.15)	0.006*	7.83 (2.25-27.27)	0.001*
Gleason score	1.12 (0.69-1.81)	0.639	1.01 (0.16-6.47)	0.991
ISUP Grade	1.10 (0.77-1.57)	0.600	0.79 (0.20-3.15)	0.741
PSA level (>20 ng/ml)	0.68 (0.22-2.09)	0.503	0.74 (0.22-2.45)	0.617
miR-133a-3p level	1.07 (0.41-2.78)	0.886	1.68 (0.45-6.25)	0.441

* ISUP: International Society of Urological Pathology, HR: hazard ratio, CI: confidence interval, PSA: Prostate-specific antigen.
