# Supplementary material for: Downregulation of miR-133a-3p promotes prostate cancer bone metastasis via activating PI3K/AKT signaling
Source: J Exp Clin Cancer Res. 2018 Jul 18;37:160. doi: 10.1186/s13046-018-0813-4 (PMC6052526; doi:10.1186/s13046-018-0813-4)
Supplement: Supplementary file 8 — Figure S2. Low expression of miR-133a-3p correlates with poor clinicopathological characteristics and progression-free survival in PCa patients. (A) miR-133a-3p expression levels in PCa tissues with different Gleason score as assessed by TCGA. (B) miR-133a-3p expression levels in PCa tissues with different tumor volume as assessed by TCGA. (C) miR-133a-3p expression levels in PCa tissues with different lymph node metastasis status as assessed by TCGA. (D) miR-133a-3p expression levels in PCa tissues with different distant metastasis status as assessed by TCGA. (E) Kaplan–Meier analysis of overall survival curves of PCa patients with high miR-133a-3p expression (n = 247) versus low miR-133a-3p expression (n = 247) as assessed by TCGA. (F) Kaplan–Meier analysis of progression-free survival curves of PCa patients with high miR-133a-3p expression (n = 228) versus low miR-133a-3p expression (n = 219) as assessed by TCGA. (PDF 247 kb) [file 13046_2018_813_MOESM8_ESM.pdf]

Table S8. Univariate and multivariate analysis of factors associated with bone metastasis free survival in 223 patients with prostate adenocarcinoma.

Characteristics	Univariate analysis		Multivariate analysis	
	HR (95% CI)	*P* values	HR (95% CI)	*P* values
Age (>62)	1.38 (0.84-2.26)	0.206	1.24 (0.73-2.09)	0.427
T classification	3.08 (1.18-5.33)	<0.001*	1.37 (0.68-2.78)	0.382
N classification	3.52 (2.14-5.77)	<0.001*	1.91 (1.09-3.35)	0.024*
Gleason score	1.95 (1.49-2.55)	<0.001*	0.49 (0.18-1.36)	0.171
ISUP Grade	1.78 (1.43-2.22)	<0.001*	2.18 (0.91-5.21)	0.080
PSA level (>20 ng/ml)	1.46 (0.88-2.43)	0.141	1.20 (0.71-2.01)	0.503
miR-133a-3p level	0.19 (0.11-0.36)	<0.001*	0.37 (0.19-0.74)	0.005*

* ISUP: International Society of Urological Pathology, HR: hazard ratio, CI: confidence interval, PSA: Prostate-specific antigen.
