# Supplementary figures and images for: Downregulation of miR-133a-3p promotes prostate cancer bone metastasis via activating PI3K/AKT signaling
Source: J Exp Clin Cancer Res. 2018 Jul 18;37:160. doi: 10.1186/s13046-018-0813-4 (PMC6052526; doi:10.1186/s13046-018-0813-4)

**Supplemental Figure 1**

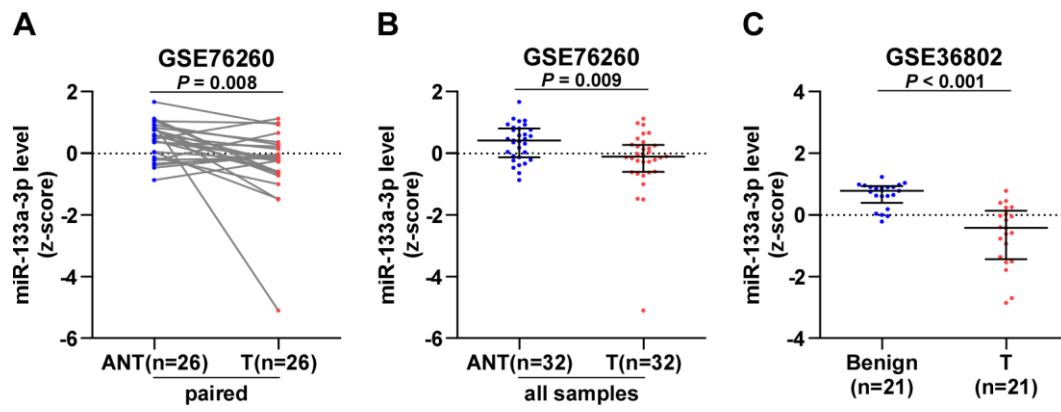

Supplement: Supplementary file 9 — Table S7. Univariate and multivariate analysis of factors associated with overall survival in 245 patients with prostate adenocarcinoma. (PDF 10 kb) [file 13046_2018_813_MOESM9_ESM.pdf]

**Supplemental Figure 2**

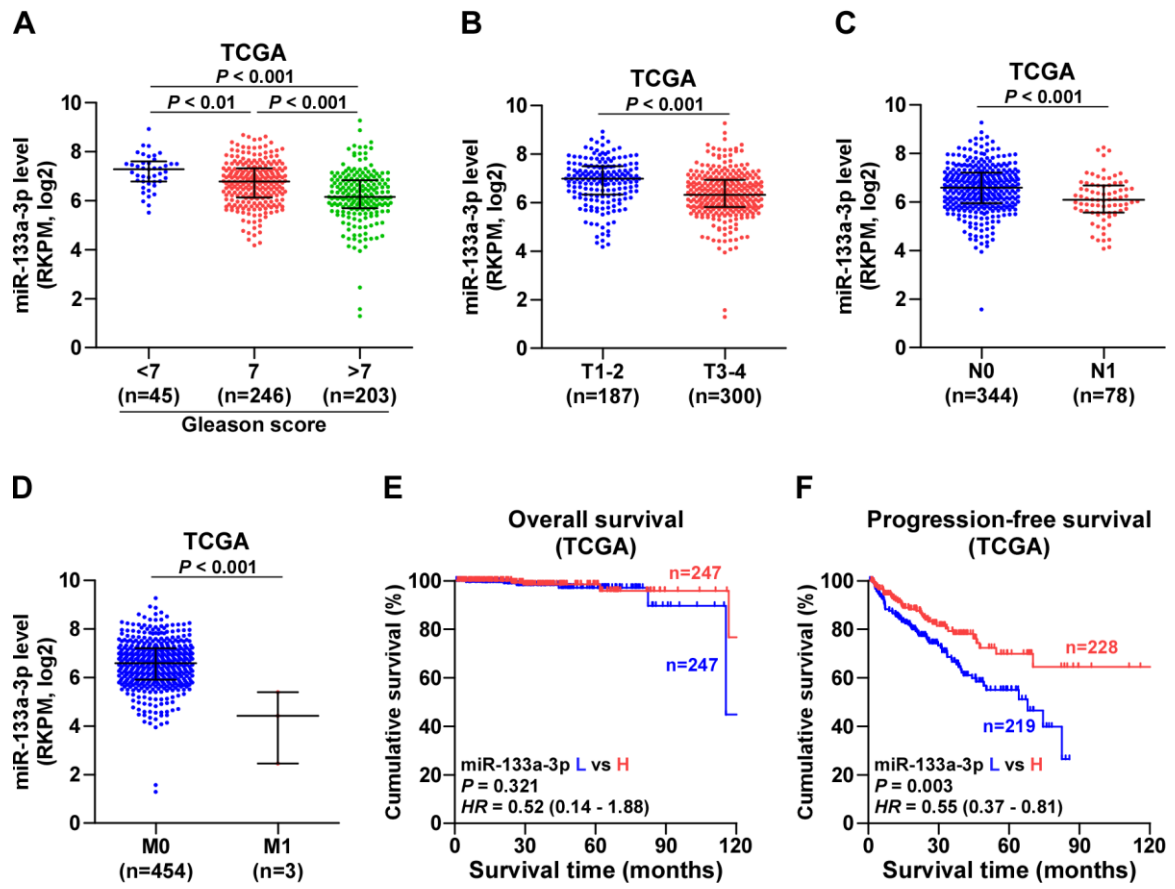

Supplement: Supplementary file 10 — Table S8. Univariate and multivariate analysis of factors associated with bone metastasis free survival in 223 patients with prostate adenocarcinoma. (PDF 10 kb) [file 13046_2018_813_MOESM10_ESM.pdf]

Supplemental Figure 3

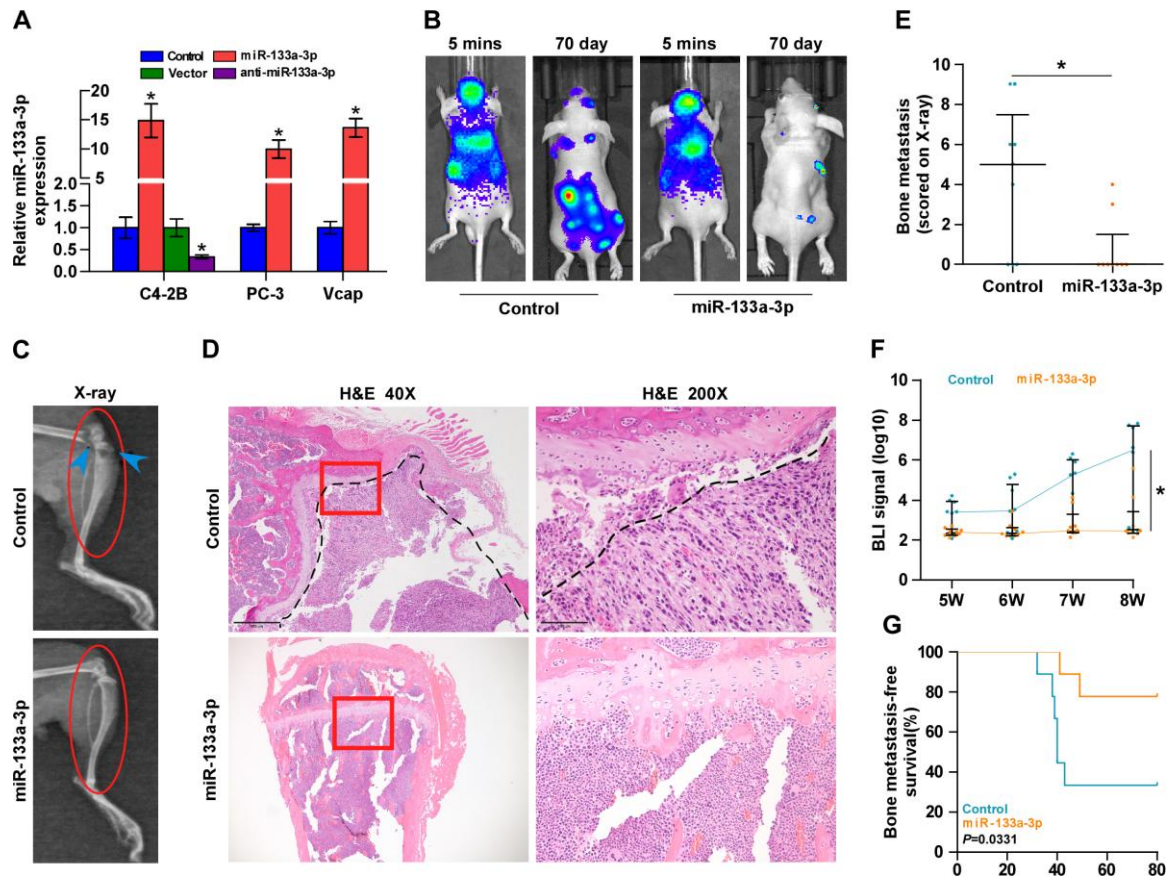

Supplement: Supplementary file 11 — Figure S3. Real-time PCR analysis of miR-133a-3p expression in the indicated PC-3, C4-2B and VCaP cells. Transcript levels were normalized by U6 expression. Error bars represent the mean ± s.d. of three independent experiments. *P < 0.05. (PDF 173 kb) [file 13046_2018_813_MOESM11_ESM.pdf]

**Supplemental Figure 4**

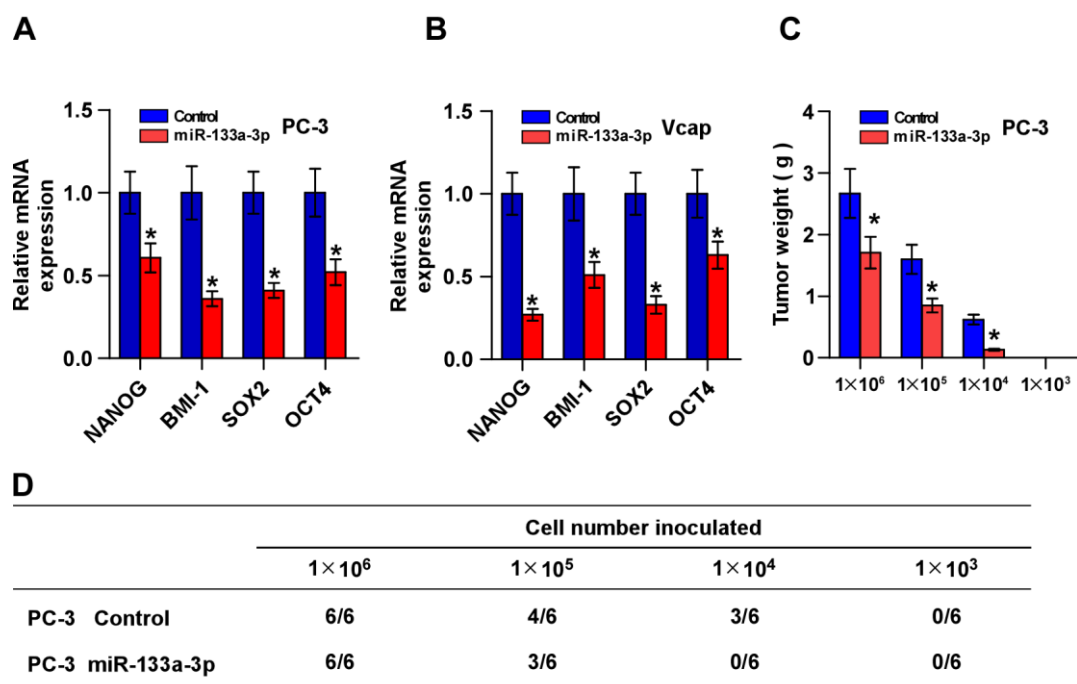

Supplement: Supplementary file 12 — Figure S4. (A and B) Real-time PCR of NANOG, BMI-1, SOX2 and OCT4 in the indicated cells. Transcript levels were normalized by U6 expression. Error bars represent the mean ± s.d. of three independent experiments. *P < 0.05. (C) Histograms show the mean tumor weights of each group. *P < 0.05. (D) The number of tumor formation initiated by different amounts of PC-3 cells in nude mice. n = 6 per group. (PDF 104 kb) [file 13046_2018_813_MOESM12_ESM.pdf]

Supplemental Figure 5

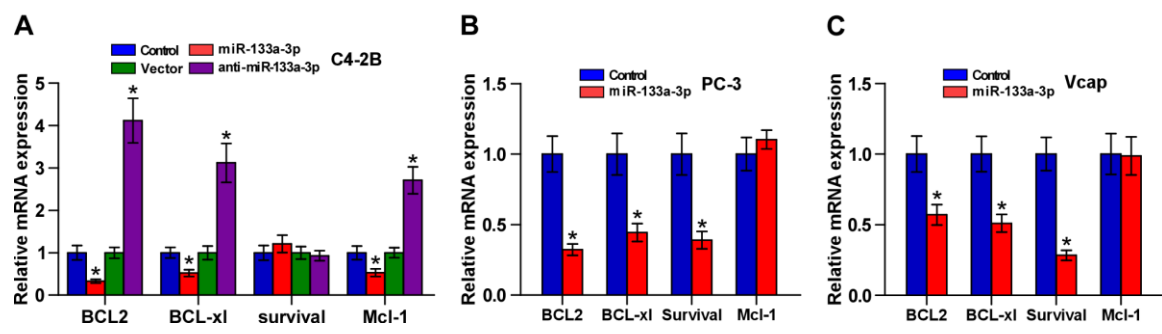

Supplement: Supplementary file 13 — Figure S5. (A-C) Real-time PCR of BCL2, BCL-xL, Survivin and Mcl-1 in the indicated cells. Transcript levels were normalized by U6 expression. Error bars represent the mean ± s.d. of three independent experiments. *P < 0.05. (PDF 72 kb) [file 13046_2018_813_MOESM13_ESM.pdf]

Supplemental Figure 6

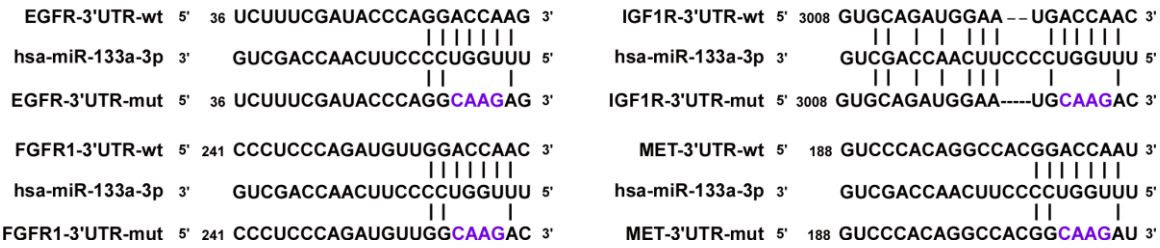

Supplement: Supplementary file 14 — Figure S6. Predicted miR-133a-3p targeting sequence and mutant sequences in 3′UTRs of EGFR, FGFR1, IGF1R and MET. (PDF 109 kb) [file 13046_2018_813_MOESM14_ESM.pdf]

Supplemental Figure 7

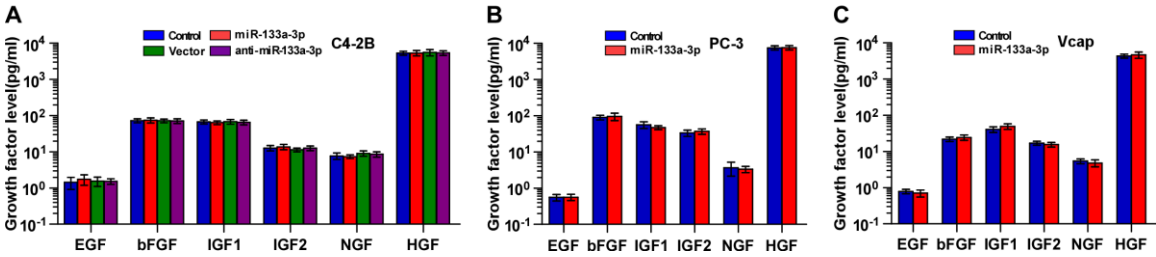

Supplement: Supplementary file 15 — Figure S7. (A-C) ELISA analysis of EGF, bFGF, IGF1, IGF2, NGF and HGF concentration in the supernatant of the indicated cells. (PDF 68 kb) [file 13046_2018_813_MOESM15_ESM.pdf]

**Supplemental Figure 8**

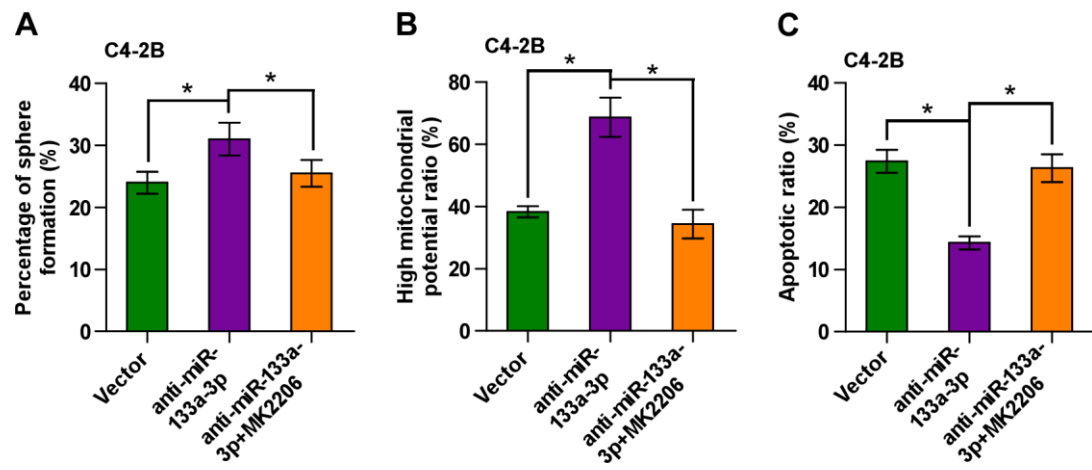

Supplement: Supplementary file 16 — Figure S8. AKT signaling was essential for the pro-tumor roles of silencing miR-133a-3p in PCa cells. (A) AKT inhibitors MK2206 (1 μM) decreased sphere formation ability in miR-133a-3p-silencing PCa cells.*P < 0.05. (B) AKT inhibitors MK2206 (1 μM) decreased the mitochondrial potential in miR-133a-3p-silencing PCa cells.*P < 0.05. (C) AKT inhibitors MK2206 (1 μM) promoted the apoptosis rate in miR-133a-3p-silencing PCa cells.*P < 0.05. (PDF 97 kb) [file 13046_2018_813_MOESM16_ESM.pdf]
